# Alternative ribosomal proteins are required for growth and morphogenesis of *Mycobacterium smegmatis* under zinc limiting conditions

**DOI:** 10.1371/journal.pone.0196300

**Published:** 2018-04-23

**Authors:** Allexa Dow, Sladjana Prisic

**Affiliations:** Department of Microbiology, University of Hawai‛i at Mānoa, Honolulu, Hawai‛i, United States of America; University of Rochester, UNITED STATES

## Abstract

Zinc is an essential micronutrient required for proper structure and function of many proteins. Bacteria regularly encounter zinc depletion and have evolved diverse mechanisms to continue growth when zinc is limited, including the expression of zinc-independent paralogs of zinc-binding proteins. Mycobacteria have a conserved operon encoding four zinc-independent alternative ribosomal proteins (AltRPs) that are expressed when zinc is depleted. It is unknown if mycobacterial AltRPs replace their primary paralogs in the ribosome and maintain protein synthesis under zinc-limited conditions, and if such replacements contribute to their physiology. This study shows that AltRPs from *Mycobacterium smegmatis* are essential for growth when zinc ion is scarce. Specifically, the deletion mutant of this operon (Δ*altRP*) is unable to grow in media containing a high-affinity zinc chelator, while growth of the wild type strain is unaffected under the same conditions. However, when zinc is gradually depleted during growth in zinc-limited medium, the Δ*altRP* mutant maintains the same growth rate as seen for the wild type strain. In contrast to *M*. *smegmatis* grown with sufficient zinc supplementation that forms shorter cells when transitioning from logarithmic to stationary phase, *M*. *smegmatis* deficient for zinc elongates after the expression of AltRPs in late logarithmic phase. These zinc-depleted bacteria also exhibit a remarkable morphology characterized by a condensed chromosome, increased number of polyphosphate granules, and distinct appearance of lipid bodies and the cell wall compared to the zinc-replete cells. However, the Δ*altRP* cells fail to elongate and transition into the zinc-limited morphotype, resembling the wild type zinc-replete bacteria instead. Therefore, the *altRP* operon in *M*. *smegmatis* has a vital role in continuation of growth when zinc is scarce and in triggering specific morphogenesis during the adaptation to zinc limitation, suggesting that AltRPs can functionally replace their zinc-dependent paralogs, but also contribute to mycobacterial physiology in a unique way.

## Introduction

Zinc (Zn^2+^), an essential micronutrient and cofactor to a myriad of proteins supporting basic cellular processes, is scantly available to many bacteria, from soil biota to pathogens within the host. To maintain essential cellular processes when Zn^2+^ ion is scarce, bacteria have evolved numerous survival mechanisms including high-affinity Zn^2+^ import systems, mobilization of cellular Zn^2+^ reserves, and replacement of certain Zn^2+^-binding proteins with alternative Zn^2+^-independent paralogs that can perform the same or similar function [1]. Genes encoding proteins involved in Zn^2+^ starvation are often repressed by the transcriptional regulator Zur (zinc uptake regulator) that forms a complex with Zn^2+^ (Zur/Zn^2+^), and de-repressed when Zn^2+^ levels reach sub-fM concentrations and the Zur/Zn^2+^ complex dissociates [2,3]. Along with high affinity Zn^2+^ import systems, Zn^2+^-independent alternative ribosomal proteins (AltRPs) are a common feature of bacterial Zur regulons [2].

Eight most commonly found zinc-independent AltRPs in prokaryotic genomes are predicted to replace their paralogous primary ribosomal proteins (PrimRPs) in both the small, 30S, (S4, S14, S18) and large, 50S, (L28, L31, L32, L33, L36) ribosomal subunits [4]. More than half of sequenced prokaryotic genomes contain at least one of these highly divergent Zn^2+^-independent AltRPs that have lost most, if not all, cysteine residues required for Zn^2+^-binding [4]. Sequence similarity of these highly divergent AltRPs is often higher amongst homologs in different species than to the PrimRP sequence within the same species, indicating the distribution of those AltRP sequences is likely the result of ancient horizontal gene transfer events followed by lineage specific evolution [5]. Operons containing ribosomal proteins and rRNA are tightly coordinated and constitute one of the most conserved super-operons in prokaryotes [6], while Zur-regulated AltRPs are removed from this selective pressure and have therefore experienced different evolutionary trajectories from both their PrimRPs and from the same AltRP in another bacterium. This separation of regulation and structure of PrimRPs *vs*. AltRPs along with the widespread distribution of AltRPs throughout prokaryotic genomes raises the possibility that AltRPs could have functionally evolved to fill specific roles in different bacterial lineages.

Direct investigations regarding AltRPs and their contribution to bacterial physiology have been explored in just a few bacteria, including *Bacillus subtilis* [7–9] and *Streptomyces coelicolor* [10], despite the fact that AltRPs are part of Zur-regulons in many bacteria, including important human pathogens [2,4]. In fact, transcriptomics studies have shown upregulation of genes encoding AltRPs in persistent/dormant populations of *Mycobacterium tuberculosis* found in human sputum and in a mouse model of infection [11,12], but their role in pathogenesis and mycobacterial physiology is not known. In *B*. *subtilis* the surface-bound AltRP L31-2 can directly liberate intracellular Zn^2+^ stored in ribosomes by displacing the non-essential Zn^2+^-containing L31-1 at the ribosomal surface, thus serving as a direct source of intracellular zinc mobilization when zinc is limited [7,13]. On the other hand, S14-2 offers a fail-safe mechanism during Zn^2+^ depletion to functionally replace essential core protein S14-1, which is presumably inactive when Zn^2+^ is unavailable [8]. These two mechanisms are regulated by sequential Zur-directed de-repression of genes encoding L31-2 and L33-2, followed by de-repression of the S14-2 gene when Zn^2+^ is further depleted, indicating precise control over AltRP expression with regards to Zn^2+^ availability in *B*. *subtilis* [14]. There is no evidence that mycobacterial AltRPs have functional roles similar to those found for *B*. *subtilis*.

**Fig 1:**
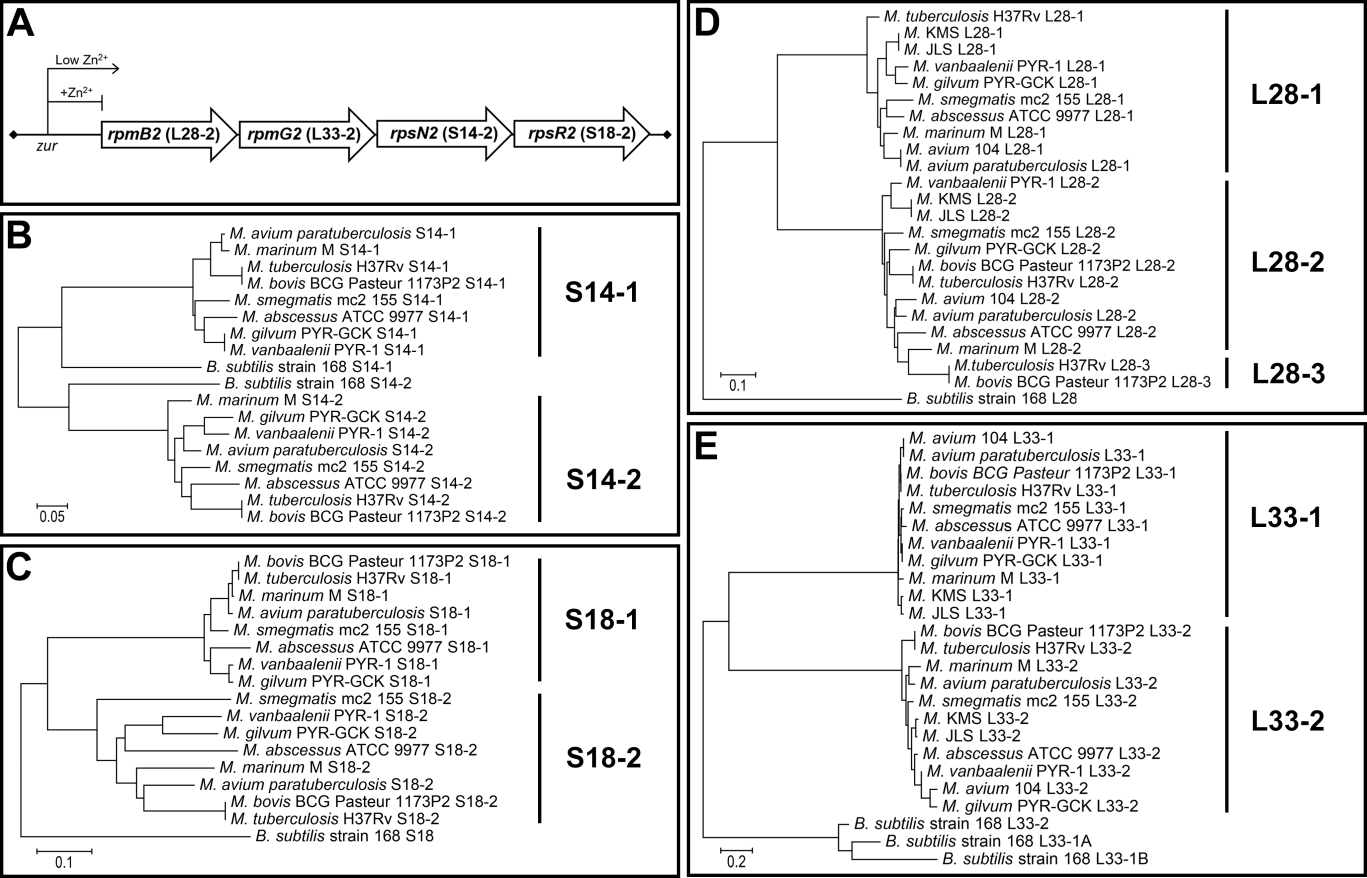
AltRPs in mycobacteria. **(A)** Organization of the conserved mycobacterial *altRP* operon. **(B-E)** Phylogenetic trees comparing protein sequence of PrimRP-AltRP pairs for the four AltRPs from the mycobacterial *altRP* operon and their PrimRP paralogs. Ribosomal proteins, and when applicable alternative copies of ribosomal proteins from *B*. *subtilis* are shown for comparison. PrimRPs have the suffix “-1” while alternative versions have the suffix “-2”. In some cases, there are additional AltRPs found outside of the *altRP* operon that are designated with the suffix “-3”. Scale bars are in units of amino acid substitutions per site.

In contrast to *B*. *subtilis* where AltRP-encoding genes are separately expressed under independent Zur-regulated promoters, mycobacteria possess a highly conserved operon, *altRP*, containing four genes encoding S14-2, S18-2, L28-2 and L33-2 AltRPs [4] (Fig 1A). As this operon is controlled by Zur, expression of AltRPs is triggered by Zn^2+^ depletion [15,16]. Changes in Zn^2+^ concentration may be an important signal for bacteria. For example, pathogenic bacteria experience a dramatic decrease in Zn^2+^ availability during the infection, due to the recruitment of neutrophils and their Zn^2+^ and manganese-binding protein calprotectin to the site of infection [17]. While growth of many bacterial and fungal pathogens is successfully controlled by calprotectin [18], *M*. *tuberculosis* can tolerate high levels of this neutrophil derived protein [15], indicating that it has adapted to survive severe Zn^2+^ and manganese depletion found in calprotectin-rich granulomas, and AltRPs may have a role in this adaptation.

The conserved nature of the mycobacterial *altRP* operon indicates that *M*. *tuberculosis*, the deadliest infectious agent and the most successful human pathogen, and related nontuberculous mycobacteria, which increasingly burden human health, share similar adaptive mechanisms to tolerate Zn^2+^ depletion. Considering the paucity of studies on the physiological response to Zn^2+^ depletion in bacteria in general, and the unknown function of the conserved *altRP* operon in mycobacteria, we set out to investigate the effects of Zn^2+^ depletion and AltRP expression on growth and morphology in the model mycobacterium, *M*. *smegmatis*. Using a deletion mutant strain of the *altRP* operon (Δ*altRP*) made it possible to show that AltRPs in *M*. *smegmatis* are essential for survival in severely Zn^2+^-depleted conditions, but also contribute to bacterial physiology during gradual Zn^2+^ depletion.

## Materials and methods

### Sequence analysis of mycobacterial AltRPs

Mycobacterial ribosomal protein sequences chosen for sequence analysis were selected based on the mycobacterial genomes previously identified to have alternative ribosomal proteins [4] (S1 Table). For simplicity, only one strain per species is shown. Protein sequences were downloaded from UniProt database. Due to the inconsistency in annotations for primary and alternative proteins, we used the sequence alignments and the presence of Cys-rich Zn^2+^-binding motifs (CxxC) to ensure the proper annotations for Prim/Alt protein versions (S1 Fig). Phylogenetic trees were generated using the Neighbor-Joining method [19] and were rooted using the corresponding ribosomal protein sequences of *B*. *subtilis* and, when present, AltRP protein sequences from *B*. *subtilis* were also included. Trees are drawn to scale and evolutionary distances are calculated using the Poison correction method [20]. Evolutionary analyses were conducted in MEGA7 [21].

### Media

All chemicals were purchased from Fisher Scientific, unless otherwise noted. For strain construction and maintenance, *M*. *smegmatis* was grown on agar plates with Middlebrook 7H9 (Difco) supplemented with ADC (0.5% albumin, 0.2% glucose, 0.085% NaCl) or liquid 7H9/ADC-T (with 0.05% Tween 80). Chemically defined Sauton’s medium (0.05% KH_2_PO_4_, 0.05% MgSO_4_7H_2_O, 0.2% citric acid, 0.005% ferric ammonium citrate, 6% glycerol, 0.4% Asparagine, 0.05% Tween 80, pH 7.4) was used for all growth experiments. For Zn^2+^- replete medium (ZRM), ZnSO_4_ was added to medium at 6 μM final concentration. Zn^2+^ limitation/deficiency in batch cultures was achieved either by omitting ZnSO_4_ in Sauton’s Zn^2+^-limited medium (ZLM) or by adding a Zn^2+^- chelating agent TPEN (N,N,N′,N′-tetrakis (2-pyridinylmethyl)-1,2-ethanediamine) respectively, as indicated.

### Strains and genetic manipulation

*M*. *smegmatis* mc^2^ 155 was a gift from Dr. Robert Husson, Boston Children’s Hospital, and originates from the ATCC stock collection. A deletion mutant for the *altRP* operon (Δ*altRP*) was constructed in *M*. *smegmatis* mc^2^ 155 background as previously described [15]. In short, 1 kb flanking regions of the operon were cloned into pDONR221 vector (Life Technologies), followed by inserting a hygromycin resistance marker cassette between the flanking regions using an engineered PacI restriction site. The construct was then transferred into pDONR1351 plasmid, a vector derived from pRH1351 [22] into a donor vector using Gateway conversion system (Life Technologies). Clones with double cross-over events were selected as previously described [22] and proper insertion was confirmed with PCR and sequencing. Complementation of the deletion mutant for the *altRP* operon was achieved by transforming the mutant with an integrating plasmid containing *altRP* operon and its native promoter (pMV306-P_altRP_-*altRP*), constructed by cloning the *altRP* operon with its native promoter (300 bp upstream of the start codon) into pMV306 vector [23]. Positive clones were confirmed by PCR and sequencing.

To monitor *altRP* promoter activity during growth, wild type *M*. *smegmatis* mc^2^ 155 was transformed with a plasmid carrying a fluorescent reporter, mCherry, expressed under the *altRP* promoter in an integrating vector (pMV306-P_altRP_-*mCherry*). The protein is optimized for expression in mycobacteria and was transferred from pVV16-mCherry plasmid [24]. Fluorescence of mCherry reporter strain (590 nm/635 nm) was measured in a Tecan plate reader.

### Preparation of inoculum and seeding flasks for growth

The starting inoculum used to seed flasks for growth in Sauton’s ZLM was critical for obtaining reproducible results. If the starting inoculum was too high, the cells had larger Zn^2+^ storage, affecting Zn^2+^ depletion, while very low inoculum would sometimes prevent growth. Seed cultures for all strains tested were grown fresh at the beginning of each experiment by inoculating 5 mL of 7H9/ADC-T in a 50 mL vented conical bioreactor tube (Corning, Product #: 431720) with 10 μL of glycerol stock. The tubes were laid horizontally with shaking at 120 rpm at 37°C for 18 hours. Seed cultures after 18 hours were lightly turbid (approx. OD 1) and if the cultures were visibly clumping or if the culture was too dense, they were discarded. Cells were harvested by centrifugation at 3,000x*g* and were washed three times in Sauton’s ZLM at room temperature. Washed seed cells were normalized to OD 1 and 50 μL of the normalized cell suspension was used to inoculate 50 mL of Sauton’s ZLM (1:1000 dilution factor) in a 250 mL vented flask. Cultures were left standing at room temperature for 4 hours before incubation at 37°C with shaking at 120 rpm for the remainder of the experiment.

### Colony forming units and OD measurements

Cultures were monitored for growth by measuring optical density at 600 nm (OD_600_) in a Tecan plate reader. A regression equation was used to convert OD measurements from 200 μL samples in the plate reader to standard 1 cm path length OD values obtained experimentally. Considering that the cell length and clumping may have an effect on the light scattering, cultures were also plated for enumeration of viable cells and reported as colony forming units (CFUs) per milliliter. For CFUs, the track-dilution method described previously was used, with minor modifications [25]. The method was compared and validated with the standard spread-plate method. In short, 7H9/ADC agar was poured in square Petri dishes with 6x6 grids, which can accommodate six 10-fold dilutions. Plates were dried thoroughly at 37°C before use. Ten μL of each dilution was spotted in a row on one side of the plate and then held at a 45° angle until droplets reached the other side of the plate, *i*.*e*., until droplets formed tracks. “Tracks” were allowed to dry and plates were incubated at 37°C for 3 days for colony enumeration. Each strain was tested in biological duplicate and technical duplicates were made for each plate. The average and standard deviation of both technical and biological duplicates are reported.

### Measuring cell length

Differential interference contrast (DIC) light microscopy was used to capture images of cells throughout growth and the cell length was measured from the digital images using ImageJ [26]. Digital images were recorded from wet-mounts at 1000X magnification on an Olympus BX-51 upright compound light microscope using a 100X oil immersion lens and an Optronics Macrofire SP CCD camera. A calibration slide was also recorded at 1000X magnification and was used to convert pixel measurements to micrometers using ImageJ. Cultures were grown in biological duplicate and both replicates were visualized but since variation in cell length between replicates was never observed, analysis of cell length was reported for one of the biological replicates. Average cell length for each treatment was obtained from measurements of 100 cells that were completely planktonic (not associated with clumps) and were not “snapping” (*i*.*e*., were not in the process of cell division). Cells from 5–10 different fields of view (*i*.*e*., different images) for each treatment were used to obtain the 100 cells used for analysis. Cell lengths were corroborated via measuring ultrathin cross-sections of fixed cells using TEM, but due to the paucity of samples aligned parallel to the plane of cross-sectioning (especially for elongated cells) TEM derived cell lengths were not used for analysis. Student’s *t*-test (*p*-value <0.05) was used to determine whether average cell lengths between strains were significantly different than one another.

### Transmission electron microscopy (TEM)

Five milliliters of cells were harvested in stationary phase via centrifugation at 3,000x*g* for fixation and resin embedding for TEM analysis. Harvested cells were transferred to 1.5 mL microcentrifuge tubes and fixed with 4% glutaraldehyde in 0.1 M sodium cacodylate buffer (pH 7.4) overnight at 4°C. Fixed cell pellets were washed twice in cacodylate buffer, 20 minutes each and post fixed with 1% OsO_4_ in 0.1 M cacodylate buffer for 1 hour at room temperature. Samples were dehydrated in a graded ethanol series (30%, 50%, 70%, 85%, 95%, 100%) with two changes per ethanol concentration of 5 minutes each followed by 3 changes for 5 minutes each in 100% ethanol. Anhydrous cell pellets were substituted with propylene oxide, 3 changes of 10 minutes each and infiltrated with 1:1 mix of propylene oxide and LX112 epoxy resin overnight at room temperature. Resin embedded cell pellets were immersed twice in fresh 100% LX112 epoxy resin for 2 hours each and finally placed in molds to polymerize at 60°C for 2–3 days. Ultrathin sections (60–80 nm) were obtained on an RMC Powertome ultramicrotome and double stained with uranyl acetate and lead citrate before visualization. For whole mounts, 4 μL of bacterial cultures was dropped onto glow-discharged, carbon-coated, formvar-coated 200 mesh copper grids for 45 seconds. Cultures were wicked off the grids, washed with 10 μL distilled water and stained with 4 μL of 1% uranyl acetate for 45 seconds, followed by a final water wash and the grids were visualized immediately. Stained and grid-mounted ultrathin sections and whole mounts were loaded into a 100 kV Hitachi HT7700 transmission electron microscope and an AMT XR-41 2048x2048 pixel bottom-mount CCD camera was used for acquisition of high-resolution images.

## Results

### The mycobacterial *altRP* operon encodes four Zn^2+^-independent alternative ribosomal proteins and is conserved among mycobacteria

The Zur-regulon in *M*. *smegmatis* is relatively small, containing only 15 genes including *zur* itself, zinc ABC transporters, chaperones, and five AltRPs, four of which are in the *altRP* operon (S2 Table) (Fig 1A) [27]. Twice as many genes are under control by Zur in *M*. *tuberculosis* [16], although many of these additional proteins do not have homologues in *M*. *smegmatis*, likely signifying the role of Zn^2+^ concentration in the context of infection. The *altRP* operon is conserved in both pathogenic and non-pathogenic mycobacteria, indicating that expression of AltRPs is likely a fundamental mechanism used by mycobacteria in response to Zn^2+^ depletion.

Mycobacterial genomes usually contain 5–6 AltRPs, which is among the highest number of AltRPs seen in bacteria [4]. The *altRP* operon occurs in all mycobacteria, with the exception of *M*. *leprae* which has undergone a significant genome reduction and lacks any AltRPs, and strains of *M*. *avium* which have a truncated Zur-regulated *altRP* operon containing just two genes for AltRPs [4]. Additional genes for other AltRPs occur in some mycobacterial genomes (S1 and S2 Tables), however we suspect that the AltRPs outside of the *altRP* operon are not expressed or are quickly degraded (*e*.*g*., if they are not incorporated into ribosomes), because we were able to detect only the *altRP*-encoded AltRPs in *M*. *tuberculosis* [15] or *M*. *smegmatis* protein extracts and/or purified ribosomes using mass spectrometry (S3 Table). All four proteins encoded by the *altRP* operon are more similar to the AltRPs in other mycobacteria than to their PrimRP paralogs (Fig 1B–1E, S1 Fig), indicating their early divergence from their Zn^2+^-binding paralogs and possible specialization.

According to AltRP homology to PrimRPs and the published structure showing location of the four PrimRPs in the *M*. *smegmatis* 70S ribosome [28] (S2 Fig), two proteins expressed from the *altRP* operon are predicted to be core proteins in the small ribosomal subunit (S14-2 and S18-2) and the other two are expected to incorporate into the large ribosomal subunit (L28-2 and L33-2). The PrimRPs are in different parts of the ribosome, both in the core and at the ribosomal surface (S2 Fig), therefore, the four AltRPs that replace them may cause wide-ranging structural changes that influence various aspects of ribosome function and regulation.

### The *altRP* operon is not required for growth in standard mycobacterial media

It is expected that AltRPs will not be expressed if Zn^2+^ concentration in the growth medium is high enough to bind Zur, thus maintaining repression of the *altRP* operon. We tested if the *altRP* operon is repressed when *M*. *smegmatis* is grown in Sauton’s medium, a standard chemically defined mycobacterial growth medium containing 6 μM Zn^2+^. Here, we refer to this medium as Zn^2+^ replete medium (ZRM). In order to be able to detect expression of the *altRP* operon, a reporter strain was created by transforming wild-type *M*. *smegmatis* with an integrative plasmid carrying the fluorescent protein mCherry expressed from the Zur-regulated *altRP* promoter (pMV306-P_altRP_-*mCherry*). A similar construct previously created in *M*. *tuberculosis* has been shown to correlate fluorescence with mRNA levels and incorporation of the AltRP S18-2 into ribosomes when grown without Zn^2+^ and therefore can be reliably used for detecting Zn^2+^ depletion and expression of AltRPs [15].

**Fig 2:**
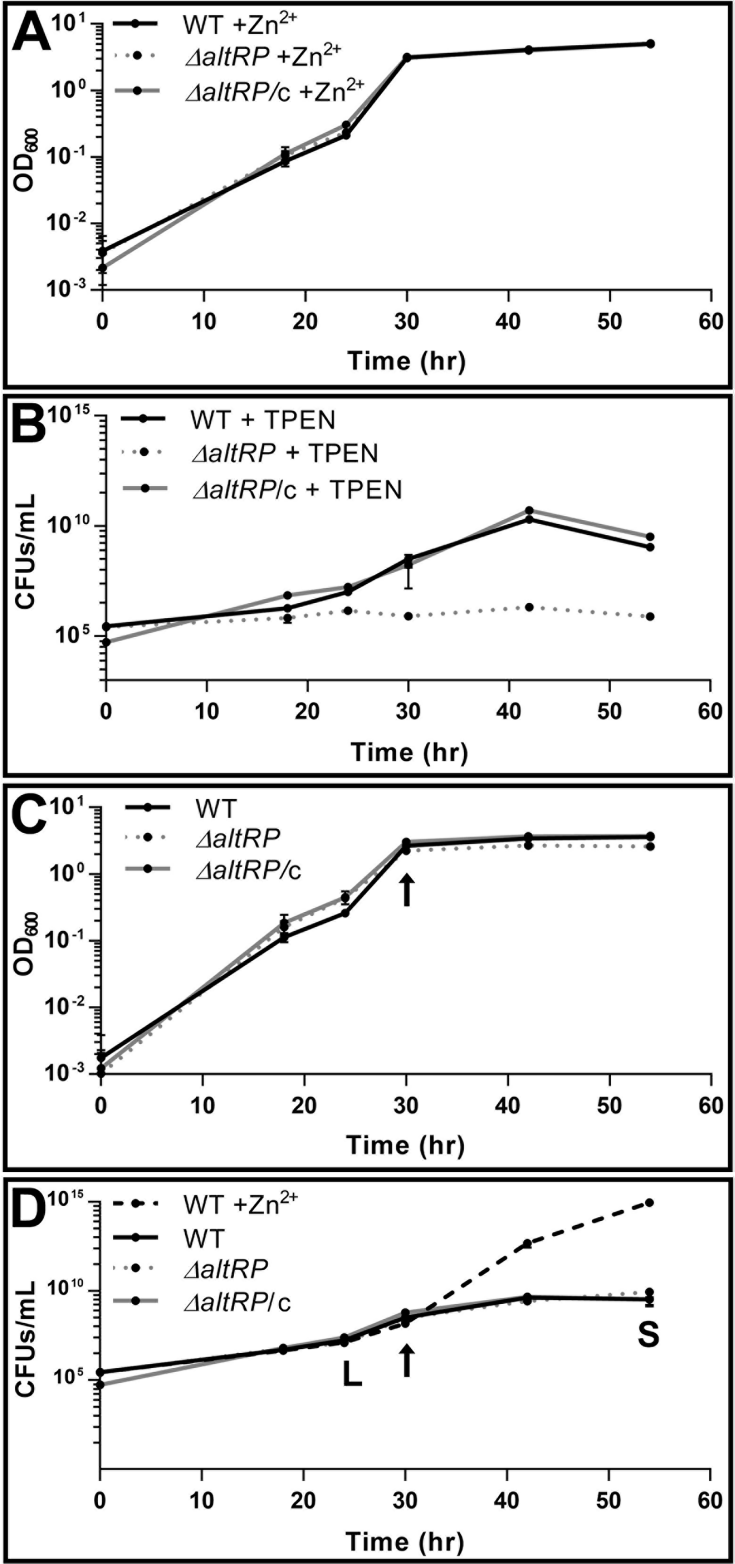
Growth of *M*. *smegmatis* strains depending on AltRPs and Zn^2+^ availability. Optical density at 600 nm (OD_600_) of WT, *ΔaltRP*, and *ΔaltRP*/c strains grown in Sauton’s medium **(A)** with 6 μM Zn^2+^ (ZRM) and **(C)** without added Zn^2+^ (ZLM). All strains were grown in parallel in biological duplicates and error bars represent the standard deviation of the average ODs between replicates. Data are representative of five independent experiments. **(B)** Colony forming units (CFUs) of the WT, *ΔaltRP*, and *ΔaltRP*/c strains grown in Sauton’s medium in the presence of 0.75 μM TPEN. (**D)** CFUs of the WT, *ΔaltRP*, and *ΔaltRP*/c strains grown in ZLM. Note that WT grown in ZRM (WT +Zn^2+^) was shown for comparison. Growth curves with CFUs were repeated twice in biological duplicate and error bars represent standard deviation of biological replicates. The letters “L” and “S” represent logarithmic and stationary growth phases, respectively. The arrow indicates the first occurrence of mCherry fluorescence detected from the P_altRP_-*mCherry* reporter strain grown in parallel with the strains presented, signifying the onset of AltRP expression. All graphs are shown with y-axes in log scale.

When *M*. *smegmatis* P_altRP_-*mCherry* reporter strain was grown in ZRM, mCherry fluorescence was not observed at any time during growth in batch culture, indicating that 6 μM Zn^2+^ is sufficient for Zur-mediated repression of the *altRP* operon (S3A Fig). As expected, all three *M*. *smegmatis* strains: wild type (WT), mutant lacking the *altRP* operon (Δ*altRP*), and its complement (Δ*altRP/*c), reached the same optical density (OD) at the same rate in this standard growth medium (Fig 2A). The same result was obtained when strains were grown in 7H9/ADC, another commonly used mycobacterial medium which contains 6 μM Zn^2+^, confirming that the *altRP* operon is not required for growth when Zn^2+^ is abundant.

### The *altRP* operon is required for growth in the presence of a Zn^2+^ chelator

AltRPs are required for survival of *M*. *smegmatis* in severely Zn^2+^-depleted environments, *i*.*e*., when grown in Sauton’s medium prepared without adding Zn^2+^ (referred to Zn^2+^ limited medium, ZLM) and in the presence of a chelating agent TPEN that binds trace amounts of Zn^2+^. At the maximum concentration of TPEN that permitted growth of WT and Δ*altRP/c* (0.75 μM), the Δ*altRP* mutant failed to grow beyond a few rounds of cell division (Fig 2B, S4 Fig). TPEN is a cell permeable divalent metal chelator that is often considered to be “Zn^2+^-specific”, however it has also been reported to from stable complexes with Fe^2+^ [29]. Indeed, *E*. *coli* transcriptional response to TPEN includes upregulation of both Zur (Zn^2+^) and Fur (Fe^2+^) regulated genes, albeit in minimal media with limiting amounts of Zn^2+^ and Fe^2+^ micronutrients [29]. ZLM used here has Fe^2+^ added at one-hundred times greater molar ratio than the amount of TPEN used, so it is unlikely that Fe^2+^ depletion contributed to the growth retardation of the mutant in ZLM beyond the effects of Zn^2+^ depletion. To ensure this assumption, we tested the ability of Zn^2+^ to rescue the growth of the Δ*altRP* mutant in ZLM with added TPEN. As expected, addition of equimolar amount of Zn^2+^ to ZLM with 0.75 μM TPEN rescued growth of the Δ*altRP* mutant (S4 Fig). The finding that Zn^2+^ can rescue growth of the Δ*altRP* mutant in the presence of TPEN indicates that AltRPs allow WT and Δ*altRP/c*, *i*.*e*., AltRP-expressing strains, to overcome this TPEN-induced Zn^2+^ deficiency.

### The *altRP* operon is not required for growth when Zn^2+^ is gradually depleted

Growth in batch culture has been demonstrated to be optimal for studying the physiological response to Zn^2+^ depletion in bacteria, as the culture gradually becomes Zn^2+^ limited, while chelating agents, such as TPEN, may interfere with normal growth by sequestering and stripping metal ions from biologically active sites [30]. When Zn^2+^ was omitted from Sauton’s growth medium (ZLM), mCherry fluorescence was observed from the P_altRP_-*mCherry* reporter strain during logarithmic growth (S3A Fig, depicted by the arrow in Fig 2C), and the presence of S18-2 mRNA (one of the AltRPs) was confirmed in both AltRP-expressing strains (WT and Δ*altRP*/c) by qRT-PCR (S5 Fig). *M*. *smegmatis* strains grew well in ZLM, but both WT and Δ*altRP*/c had decreased OD compared to the growth in ZRM; the Δ*altRP* strain appeared to have slightly reduced OD (Fig 2C). However, this difference in OD between the strains grown in ZLM was likely due to differences in clumping and/or cell size (see below) and not due to the difference in cell numbers, as there was no significant difference between Δ*altRP* and the other two strains, WT and Δ*altRP*/c, in number of colony forming units (CFUs) (Fig 2D). All strains grown in ZLM showed the same decrease in CFUs when compared to growth in ZRM, indicating that Zn^2+^ limitation slows down cell division regardless of presence or absence of AltRPs. Therefore, even limiting amount of Zn^2+^ was sufficient to allow for proper functioning of PrimRP-containing ribosomes in the Δ*altRP* mutant, as judging from CFUs, the Δ*altRP* mutant showed no sign of growth impairment in ZLM compared to WT, demonstrating that AltRPs are not required for growth when Zn^2+^ is gradually depleted.

Growth rates of *M*. *smegmatis* in ZRM and ZLM were identical until the onset of AltRP expression, after which the Zn^2+^-replete culture followed exponential cell growth and the Zn^2+^-limited culture followed a linear slope obtaining a much lower final cell density (Fig 2D), similar to previous reports for Zn^2+^-starved *Escherichia coli* batch cultures [30]. This observation indicates that reduced cell growth as a result of Zn^2+^ limitation occurs soon after de-repression of the Zur regulon. However, CFUs increased nearly two orders of magnitude after onset of mCherry fluorescence (S3A Fig, arrow in Fig 2D) and before the stationary phase, indicating that cells continued to divide after induction of AltRP expression. We previously showed that ribosomes isolated from *M*. *tuberculosis* grown in ZLM contain AltRPs [15]. Similarly, mass spectrometry analysis of purified ribosomes from WT *M*. *smegmatis* grown to stationary phase in ZLM had all four *altRP*-encoded AltRPs (underlined in S3 Table). PrimRPs were also identified, although with lower spectral count, which may indicate lower abundance of PrimRPs *vs*. AltRPs (S3 Table). This result showed that both PrimRP and AltRP-containing ribosomes were present in stationary phase when *M*. *smegmatis* was grown in ZLM. Together, these data indicate that AltRPs incorporate into ribosomes during growth, but their incorporation does not have any effect on the overall growth rate of the culture when Zn^2+^ is gradually depleted.

### Zn^2+^ availability and presence of AltRPs influence colony morphology

**Fig 3:**
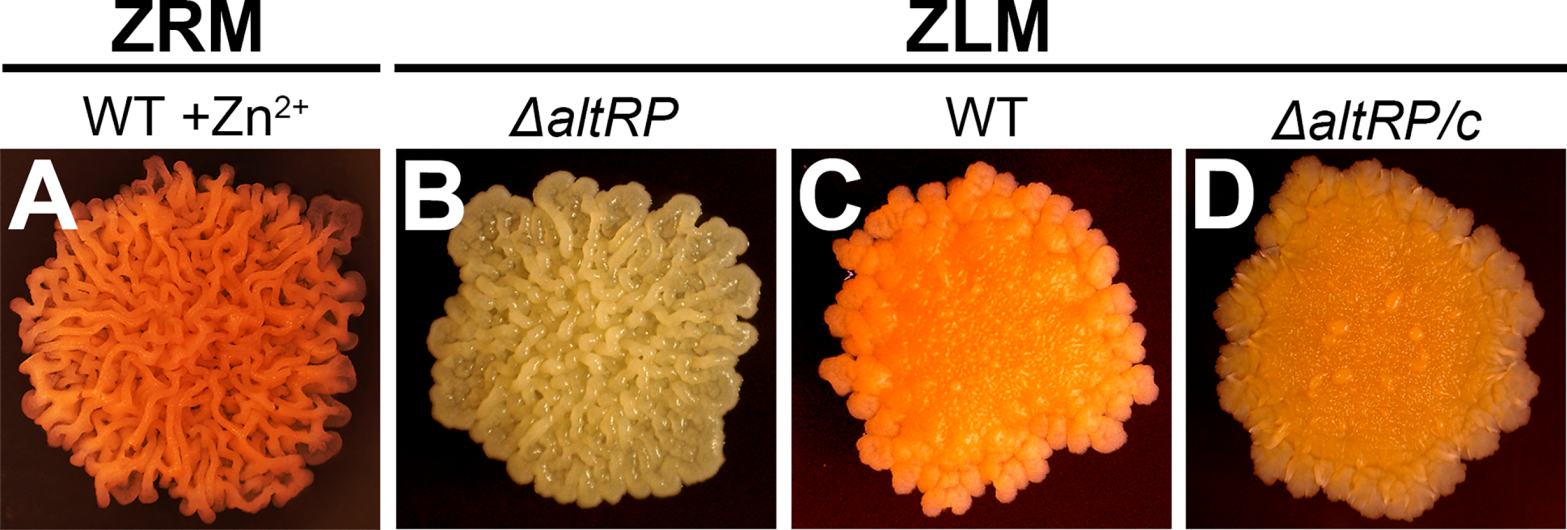
Colony morphology of *M*. *smegmatis*. **(A)** Wild type (WT) strain grown on Sauton’s agar with 6 μM Zn^2+^ (ZRM) and **(B)** *ΔaltRP*, **(C)** WT, and **(D)** *ΔaltRP/c* on Sauton’s agar without added Zn^2+^ (ZLM). Colonies are all approximately 1cm in diameter and were grown at room temperature with light for one week after initial incubation at 37°C.

In order to explore how *M*. *smegmatis* responds to Zn^2+^-replete *vs*. Zn^2+^-limiting medium, we further analyzed growth and morphology on solid medium. Similar to growth in liquid cultures, all three strains grew the same on standard Sauton’s or 7H9/ADC agar plates, but the Δ*altRP* mutant failed to grow if 0.75 μM TPEN was added to agar plates. As in liquid ZLM, Zn^2+^ depletion could be achieved on solid Sauton’s agar medium without Zn^2+^, as evidenced by the fact that the P_altRP_-*mCherry* reporter strain had pink colonies signifying AltRP expression (S3B and S3C Fig). Although all three strains grew on solid medium at the same rate, colony morphology of WT *M*. *smegmatis* grown on ZRM and ZLM were clearly distinct (Fig 3). WT colonies grown on ZRM plates were wrinkled and elevated, which is a typical appearance for *M*. *smegmatis* colonies (Fig 3A). However, WT (and Δ*altRP/*c) colonies grown on ZLM plates lacked the three-dimensional structure and had an overall flat and waxy appearance compared to Zn^2+^-replete colonies (Fig 3C and 3D). The Δ*altRP* mutant grown on ZLM exhibited a colony structure similar to WT grown on ZRM, indicating the involvement of AltRPs in changes in colony morphology that are triggered by limited Zn^2+^availability (Fig 3B). Interestingly, the Δ*altRP* mutant lacked orange pigmentation (associated with carotenoid production [31]) that was observed in all other strains grown in light, pointing at a possible role of AltRPs in pigment (carotenoid) production. Of note, the lack of pigment production in the Δ*altRP* strain was also apparent in liquid cultures in late stationary phase, WT and Δ*altRP/c* strains appeared light orange/brown and the Δ*altRP* mutant was cream colored. Distinct colony morphology observed here (Fig 3) may indicate changes in the cell wall structure caused by different Zn^2+^ availability and/or AltRP expression.

### Limited Zn^2+^ availability causes elongation of the WT but not the Δ*altRP* mutant

**Fig 4:**
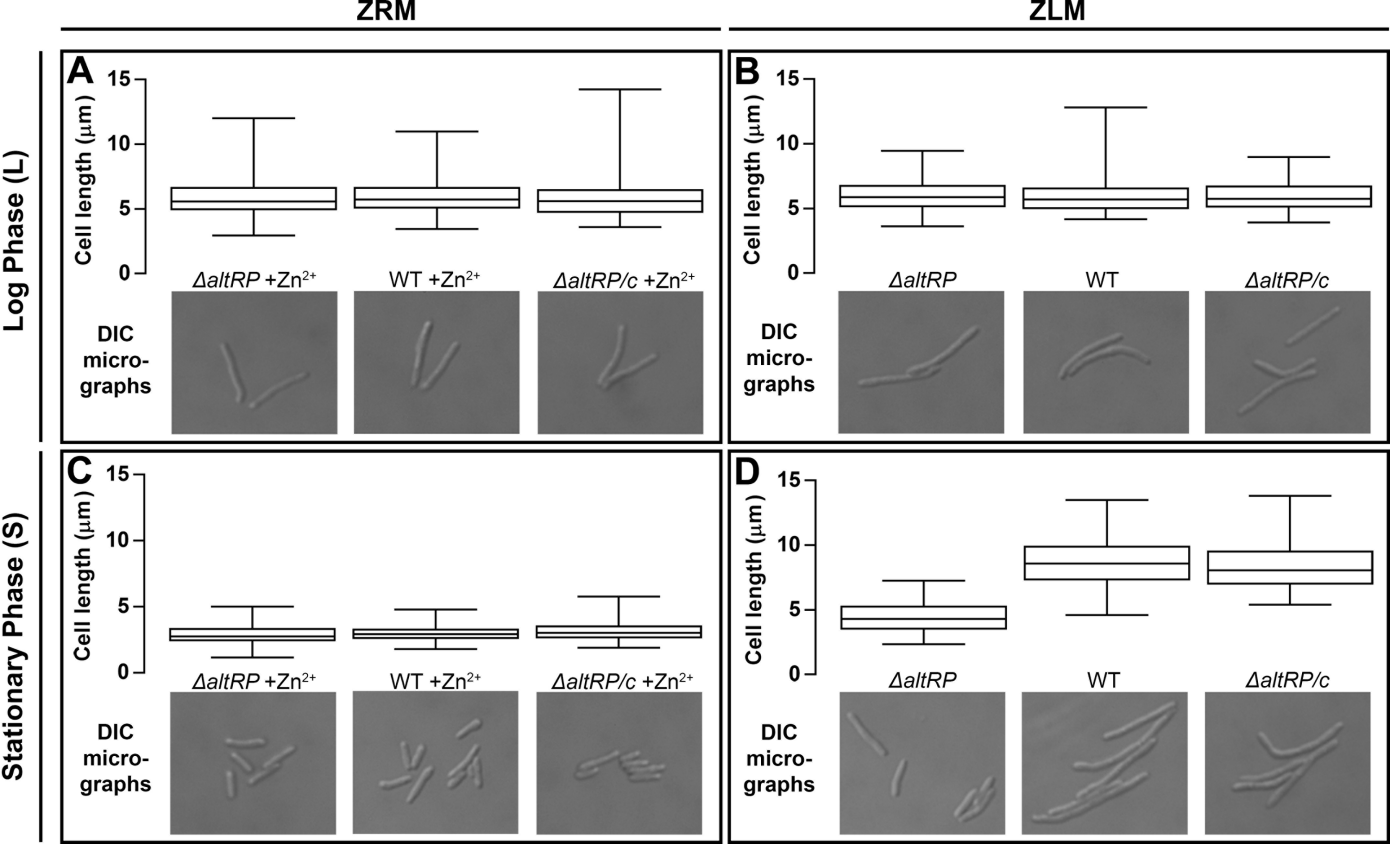
Cell length of *M*. *smegmatis* strains depending on AltRPs and Zn^2+^ availability. **(A-D)** Box and whisker plots showing the minimum, maximum, interquartile range and median cell lengths observed for WT, *ΔaltRP*, and *ΔaltRP*/c strains grown in Sauton’s medium with (ZRM) **(A,C)** and without added Zn^2+^ (ZLM) **(B,D)**. Cell length in logarithmic phase, before AltRP expression is shown in panels A and B and cell length after AltRP expression in stationary phase is shown in panels C and D. Representative DIC light micrographs are shown below the box and whisker plot for each strain at both time points. There is no statistically significant difference between average cell lengths of any strains in logarithmic phase (A,B), or between any strains at stationary phase grown in ZRM (C). There is a significant difference in the cell lengths of WT and *ΔaltRP*/c *vs*. the *ΔaltRP* mutant grown in ZLM (D). (Student’s *t*-test *p*<0.05). The data in this figure are representative of three independent experiments.

To further our understanding of the response to Zn^2+^ limitation beyond culture growth and colony morphology, we investigated cell size and morphology of *M*. *smegmatis* grown in batch culture using light microscopy. With or without added Zn^2+^, *M*. *smegmatis* cells were 6 μm long in early logarithmic phase (Fig 4A and 4B). The length of cells grown in ZRM decreased to 3.8 μm by late logarithmic phase and 2.9 μm by stationary phase (Fig 4C). This observation is consistent with other studies that have followed cell length in *M*. *smegmatis* using standard Zn^2+^-containing growth media [32]. All three strains, WT, Δ*altRP*, and Δ*altRP/c* showed the same trend when grown in ZRM, *i*.*e*., they got shorter as the culture got older (compare Fig 4A and 4C). Remarkably, while *M*. *smegmatis* cells grown in ZLM were the same size as those grown in ZRM at the early-log phase (Fig 4B), they instead grew longer, reaching an average cell length of 8.7 μm at the stationary phase (Fig 4D). Therefore, *M*. *smegmatis* cells depleted for Zn^2+^ contrast the trend previously observed in mycobacteria, *i*.*e*., they form unbranched filaments, rather than shorter bacilli, as they are reaching stationary phase.

The elongated phenotype of *M*. *smegmatis* grown under Zn^2+^- limiting conditions was unexpected, but even more surprising was that the Δ*altRP* mutant grown in ZLM not only failed to elongate, but it shortened in stationary phase, from 6 μm in early log to 4.4 μm in stationary phase, as seen for the WT grown in ZRM (Fig 4C and 4D). We continued observing cultures into late stationary phase (Day 7) at which point most bacilli became associated with clumps, making them difficult to enumerate and observe. However, the planktonic cells that were visible did not appear different from those described in early stationary phase (Fig 4), specifically they did not continue to elongate as the culture aged. Therefore, although not required for growth when Zn^2+^ is gradually depleted in batch culture, AltRPs are involved in the specific elongated cell phenotype employed during Zn^2+^ depletion.

### Elongated cells formed in response to Zn^2+^-depletion are aseptate and have distinct cell wall morphology

**Fig 5:**
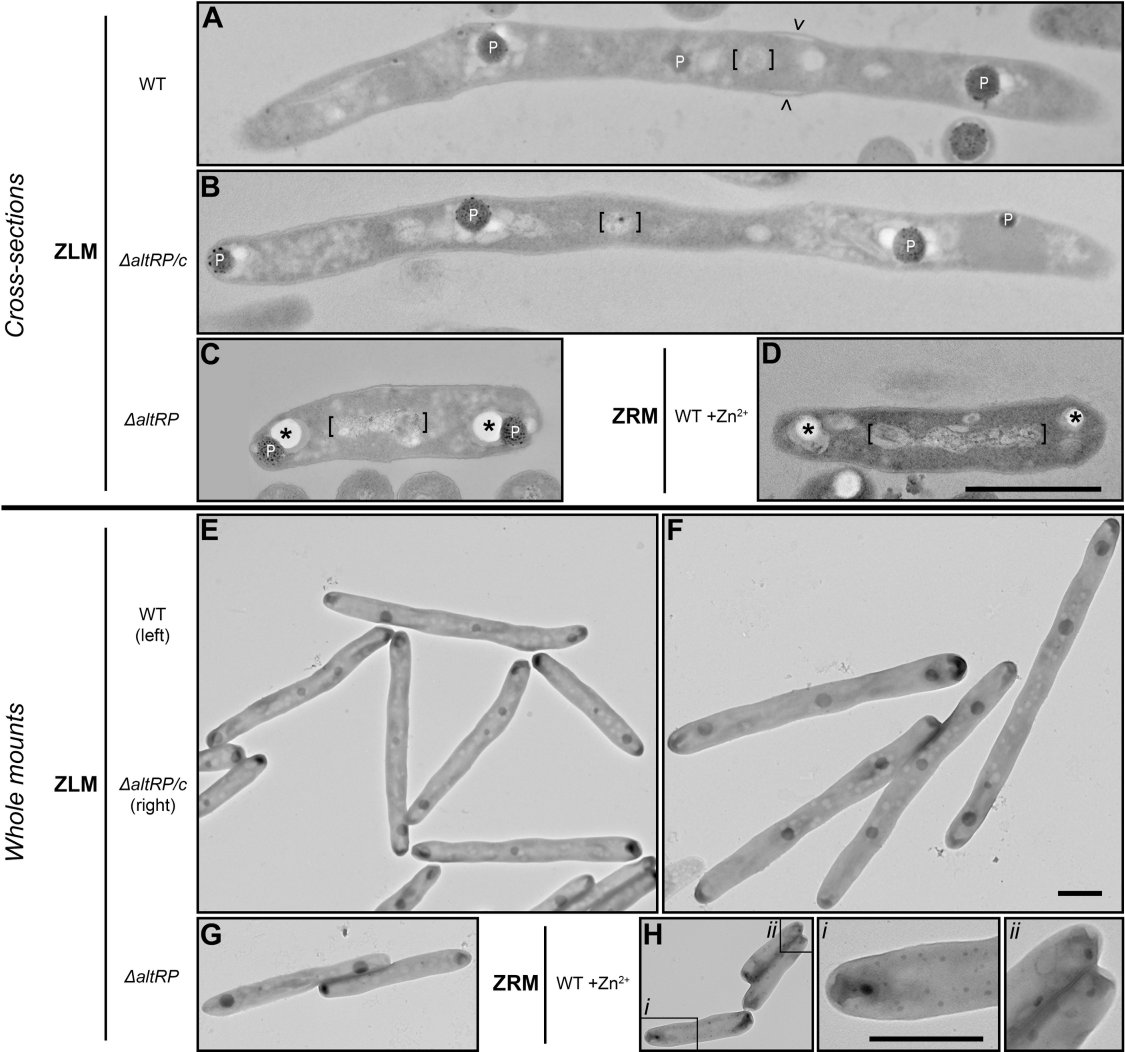
Ultrastructure of *M*. *smegmatis* exposed to Zn^2+^ depletion and in relation to AltRPs. **(A-D)** Representative TEM images depicting cross sections of cells harvested at stationary phase (corresponding to panels C and D in Fig 4). (A) WT, (B) Δ*altRP*/c, and (C) Δ*altRP* grown without added Zn^2+^ (ZLM) and (D) WT grown with Zn^2+^ supplementation (ZRM). Images are annotated as follows: polyphosphate bodies (P), asterisk indicates empty space left as an artifact of ultra-thin cross-sectioning when polyphosphate bodies were dislodged or lost altogether (see text), nucleoid region is denoted with square brackets and carets are used to define regions of the thickened cell wall. The scale bar in panel (D) is 1 μm in length and corresponds to panels (A-D) which are to the same scale. **(E-H)** Representative TEM images depicting whole mounts of cells harvested at stationary phase (corresponding to panels C and D in Fig 4). (E) WT, (F) Δ*altRP/c*, and (G) Δ*altRP* grown without added Zn^2+^ (ZLM) and (H) WT grown with Zn^2+^ supplementation (ZRM). Scale bar in panel F is 1μm in length and corresponds to panels (E-H). Panels *i* and *ii* are high magnification images that correspond to the boxed regions in panel H. Scale bar in panel *i* is 1 μm in length and corresponds to panels *i* and *ii*.

Given the differences in cell length between WT and Δ*altRP* strains and the possibility that elongated cells were actually multiple cells that fail to separate, or are arrested in cell division, as observed in other elongated forms of mycobacteria [33–36], we further investigated the ultrastructural morphology of stationary phase cells grown in ZRM and ZLM. Transmission electron microscopy (TEM) was used to visualize resin-embedded cross-sections for ultrastructure analysis as well as whole mounts of bacteria on a formvar-coated mesh copper grid for global cell analysis (Fig 5). Ultrathin cross-sections revealed that the cell wall was continuous in all strains and there was no sign of invagination or septation in the elongated WT (and Δ*altRP*/c) cells when grown in ZLM (Fig 5A and 5B). Similarly, whole mounts show that the vast majority of elongated cells were single cells and most dividing cells had a typical “snapping” appearance. Therefore, the elongation seen in *M*. *smegmatis* during Zn^2+^-depletion is not due to multiple short cells that did not separate, *i*.*e*., it is not a result of an incomplete cell division. Additionally, regions of thicker cell wall (or enlarged “periplasmic space” between the cell membrane and the cell wall) were observed in numerous cross-sections of elongated WT and Δ*altRP/c* strains grown in ZLM (carets in Fig 5A), which was never observed in the Δ*altRP* mutant grown in ZLM or WT grown in ZRM. Therefore, the AltRP-expressing morphotype of *M*. *smegmatis* (WT and Δ*altRP*/c grown in ZLM) is characterized by elongated individual cells and distinct cell wall morphology, compared to the short Zn^2+^-replete morphotype (WT grown in ZRM or lacking AltRPs).

### Zn^2+^ depletion leads to changes in distribution and properties of intracellular polyphosphate bodies

Upon examination of cross-sections it appears that the Δ*altRP* strain exhibits similar ultrastructure to the WT grown in ZRM, *i*.*e*., short cells with only two polar phosphate bodies (PPBs) per cell (Fig 5 and 5D). On the other hand, long WT (Δ*altRP/c*) cells grown in ZLM contained multiple PPBs at regularly spaced intervals (Fig 5A and 5B). Examination of the whole mounts confirmed the distribution of multiple PPBs in elongated cells grown in ZLM however also revealed a stark change in polyphosphate distribution between cells grown in ZRM vs. ZLM (Fig 5E–5H). Cells grown in ZRM had one of two distinct phenotypes: two discernable medium sized polar PPBs with numerous other, much smaller and randomly spaced PPBs giving these cells a “speckled” appearance (example in lower left of Fig 5H and inset *i*) or less apparent polar PPBs but multiple medium sized PPBs present (examples in upper right corner of Fig 5H and inset *ii*). In both cases the PPBs were numerous and small, as previously described in (Zn^2+^ replete) *M*. *smegmatis* using energy-dispersive X-ray spectroscopy coupled with transmission microscopy [37]. In contrast, coalescence of phosphate appears to occur when cells are grown in ZLM, PPBs are greatly enlarged in diameter (spanning more than half the cell width) but the total number of PPBs is substantially reduced and large PPBs accumulate at regularly spaced intervals across an elongated cell filament, and/or at the cell poles (Fig 5A and 5B).

**Fig 6:**
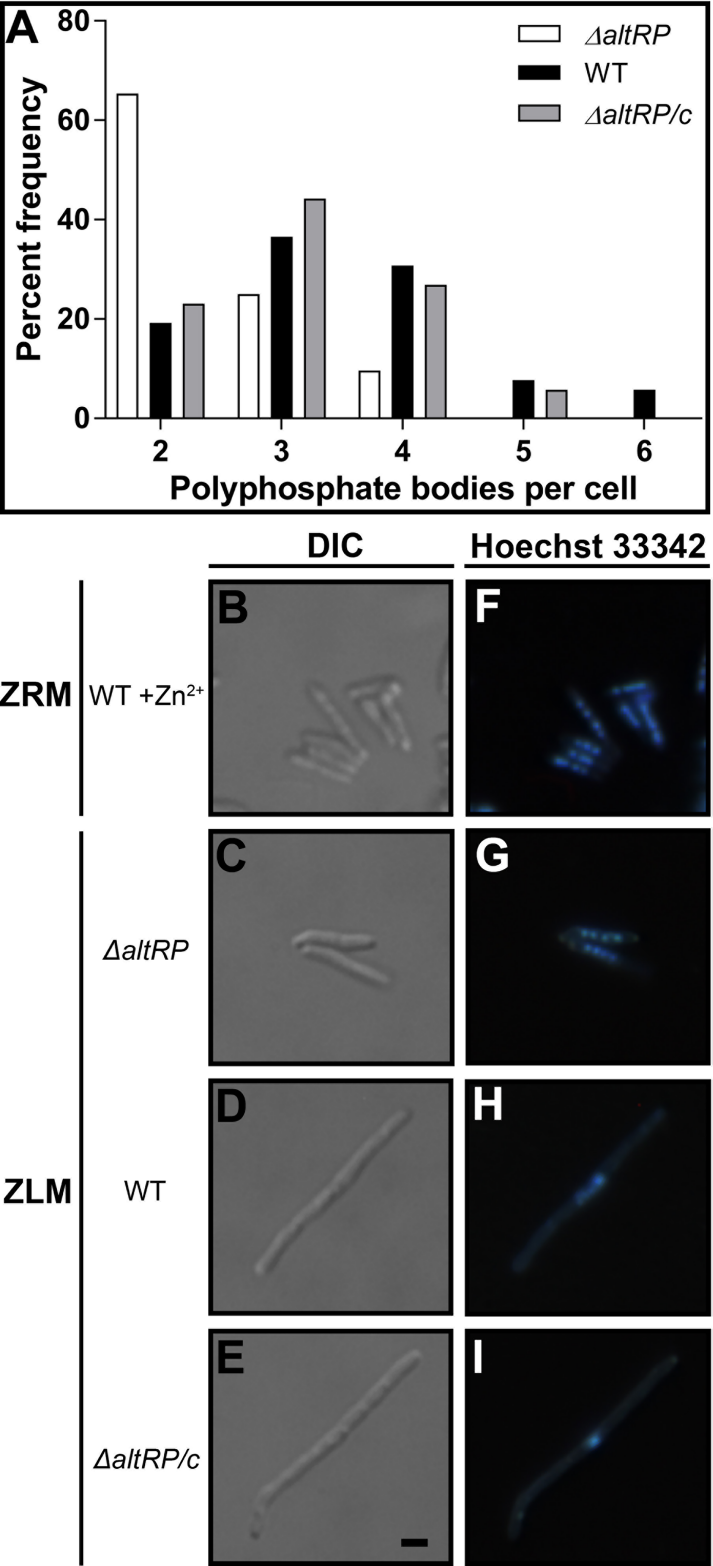
Polyphosphate body (PPB) and DNA distribution in *M*. *smegmatis* with respect to Zn^2+^ concentration and AltRP expression. **(A)** Percent frequency of the number of PPBs/cell in each strain from analysis of whole mounts with TEM. Only planktonic cells with no visible signs of septation or division were considered for analysis and 52 cells per strain were observed from at least 20 different TEM images each. **(B-E)** Bright field DIC images corresponding to panels **(F-I)** showing epifluorescent images of cells stained with the DNA dye Hoechst 33342. (B, F) WT grown with added Zn^2+^ (ZRM), (C, G) Δ*altRP*, (D, H) WT and (E, I) Δ*altRP/c* strains grown without addition of Zn^2+^ (ZLM). All images are to the same scale as represented by the scale bar in panel (E) which is 1 μm in length. Data are representative of three independent experiments.

TEM images of whole mounts showed that Zn^2+^ depletion leads to global changes in polyphosphate distribution which was not dependent on AltRPs and was observed for all strains grown in ZLM. However, considering that the Δ*altRP* strain has significantly shorter cells than AltRP-expressing strains, we were curious if the mutant would accumulate multiple large PPBs like observed in the other strains, or if it would have fewer PPBs as expected based on its size. Fig 6A shows a distribution of the percent frequency of PPBs per cell in whole mounts of WT, Δ*altRP*, and Δ*altRP/c* strains grown to stationary phase in ZLM. As observed in cross-sections, the majority (65%) of Δ*altRP* cells had 2 polar PPBs whereas WT and Δ*altRP/c* cells had on average 3–4 PPBs per cell with some cells having 5 or 6 PPBs. No changes in polyphosphate distribution were observed in cells grown in ZLM to late stationary phase (Day 7), however coalescence of polyphosphate to two larger bodies at the cell poles was observed in WT cells grown in ZRM (S6A Fig). In conclusion, Zn^2+^ depletion triggers significant redistribution of intracellular polyphosphate, leading to the accumulation of enlarged PPBs at regularly spaced intervals across an elongated cell.

When biological structures are incompletely infiltrated with resin, the poorly-embedded material is lost during ultra-thin sectioning revealing regions of empty space that appears bright white under TEM. Polyphosphate granules are known to be inefficiently infiltrated with resin during fixation, compared with other cellular structures, and are easily lost during ultrathin sectioning, leaving circular regions of empty white space in cross-sections [38]. We observed defined circular regions of empty space in cross-sections that are likely caused by the presence of PPBs in these regions of poor resin infiltration; due to the polar distribution, size and shape of the empty spaces, which correlates with PPBs observed using whole mounts described above. In Zn^2+^-replete cells, we found only two cells throughout numerous frames that retained PPBs (example in S6B Fig) and the vast majority of Zn^2+^-replete cells had empty spaces (white) where PPBs were lost (asterisks in Fig 5D). However, in Zn^2+^-depleted cells, PPBs were retained and were almost always present in their normal location in WT and Δ*altRP*/c cross sections (Fig 5A and 5B). While still present in the Δ*altRP* mutant, PPBs were often dislodged from their original space (asterisks in Fig 5C), as seen in WT grown in ZRM. These artefacts created during sample preparation may indicate that PPBs have different composition and/or interact differently with surrounding cellular components, depending on Zn^2+^ concentration and the expression of AltRPs.

### Redistribution of lipid bodies during Zn^2+^-depletion

In addition to the morphological changes described above, there was a clear difference in the appearance of intracellular lipid bodies in cells grown in ZRM and ZLM. In cross-sections, the cytoplasm of Zn^2+^-replete cells appeared dark and was contrasted by starkly defined electron translucent (light grey) lipid rich material that crescents the polar PPBs (Fig 5D). In cross-sections of Zn^2+^-depleted cells however, well organized and defined lipid bodies were diffused and widely distributed throughout the cell, and larger round lipid bodies appeared to aggregate around the PPBs in AltRP-expressing strains (Fig 5A and 5B). In the Δ*altRP* mutant, diffused and widely distributed lipid bodies were present throughout the cell, but in contrast to an aggregation of large round lipid bodies surrounding the PPBs of AltRP-expressing cells, one continuous region of well-defined lipid rich material crescents the polar PPBs, as observed in the WT grown with Zn^2+^ (Fig 5C and 5D). Whole mounts also revealed well defined, round lipid bodies throughout cells grown in ZLM (Fig 5E–5G) giving them a distinct appearance from cells grown in ZRM (Fig 5H), in which lipid bodies were not observed using this method. Aggregation of lipid bodies around PPBs is especially apparent in whole mounts of the WT and Δ*altRP*/c strains (Fig 5E and 5F). In summary, Zn^2+^ depletion in *M*. *smegmatis* leads to accumulation of clearly defined intracellular lipid bodies that tend to associate with PPBs, and the expression of AltRPs appears to influence this association.

### Central DNA condensation is part of the Zn^2+^-depletion induced morphogenesis

A striking difference in organization of the nucleoid between cells grown in ZRM and ZLM was a surprising observation in the ultrastructure analysis. When grown in ZRM, the WT had a widely distributed nucleoid spanning the middle half of the cell, with clearly visible electron dense DNA widely distributed throughout an electron transparent nucleoid region (brackets in Fig 5D). When grown in ZLM, the organization of the nucleoid in the Δ*altRP* mutant still reflected that seen in the WT grown in ZRM (brackets in Fig 5C). However, cells expressing AltRPs (WT and Δ*altRP*/c) grown in ZLM appeared to have a drastically reduced nucleoid size and exhibited centrally condensed DNA (brackets in Fig 5A and 5B). To confirm this phenotype, stationary phase cells were stained with the fluorescent dye Hoechst 33342 to visualize DNA distribution. Fluorescent images confirmed normal multi-lobed DNA distribution in all cells of the WT strain grown in ZRM and the Δ*altRP* mutant grown in ZLM (Fig 6B, 6F, 6C and 6G). Elongated WT and Δ*altRP*/c cells grown in ZLM showed centrally confined puncta of Hoechst fluorescence (Fig 6D, 6H, 6E and 6I). Both TEM and fluorescent microscopy techniques demonstrated that Zn^2+^ deficiency causes DNA condensation of *M*. *smegmatis* in an AltRP-dependent manner.

## Discussion

The conserved *altRP* operon, *i*.*e*., the Zn^2+^-independent ribosomal proteins it encodes, allows *M*. *smegmatis* to continue growing even under extremely Zn^2+^-deficient conditions. In addition to supporting growth, AltRPs are involved in remarkable morphological changes triggered by gradual Zn^2+^depletion, suggesting that changes in Zn^2+^ concentration and the resulting differential expression of AltRPs could lead to the formation of physiologically distinct bacterial subpopulations. The description of Zn^2+^-induced morphogenesis in *M*. *smegmatis* provides a valuable contribution towards the better understanding of mycobacterial physiology, especially with regards to the drastic changes in Zn^2+^ availability experienced by pathogenic mycobacteria during infection [15]. Moreover, the involvement of AltRPs in the Zn^2+^-induced morphogenesis is a novel finding; to our knowledge, this is the first time AltRPs have been reportedly involved in cell physiology beyond “rescuing” cell growth in low Zn^2+^, as previously shown for *B*. *subtilis* AltRPs [8,9].

Here, we show that *M*. *smegmatis* requires AltRPs to grow in media where Zn^2+^ is severely depleted with TPEN. However, growth of the Δ*altRP* mutant was rescued by addition of equimolar Zn^2+^ to TPEN-containing cultures, indicating that free Zn^2+^ is required for growth of the Δ*altRP* mutant. Considering that TPEN-Zn^2+^ interaction has a k_d_ in sub-fM range [39], which is several orders of magnitude lower than sub-μM k_d_ found for the PrimRP S18-1-Zn^2+^ interaction in *M*. *tuberculosis* [15], it is possible that TPEN competes for Zn^2+^ with the homologous *M*. *smegmatis* S18-1 protein (and other Zn^2+^-binding PrimRPs), thus preventing proper ribosome assembly and therefore growth of the Δ*altRP* mutant in the absence of expression of Zn^2+^-independent AltRPs. We also showed that by simply omitting Zn^2+^ from a standard chemically defined mycobacterial growth medium, AltRPs are expressed in the late logarithmic phase in *M*. *smegmatis*. The increase in cell density observed after the onset of Zn^2+^ depletion is likely due to the redistribution of intracellular Zn^2+^ with each round of cell division until cells reach a minimum Zn^2+^ concentration per cell and growth ceases. Importantly, during the period of intracellular Zn^2+^ redistribution there is no sign of slower cell division in the Δ*altRP* mutant, as would be expected if AltRPs are required to liberate Zn^2+^ from ribosomes to enable growth during Zn^2+^ limitation, or if the Δ*altRP* mutant had decreased translational capacity during Zn^2+^ limitation, due to its reliance on PrimRPs. These results suggest that AltRPs can functionally replace PrimRPs if Zn^2+^ is removed from PrimRPs or severely limited, and in these cases the expression of AltRPs is required for cell proliferation. However, cells can withstand gradual decrease of Zn^2+^ concentration during growth without AltRP expression and there was no apparent benefit to AltRP expressing strains during growth in ZLM regarding their growth rates.

Although growth and cell division of the Δ*altRP* mutant is not impaired during gradual Zn^2+^ depletion, it fails to completely undergo morphogenesis caused by Zn^2+^ limitation. WT, *i*.*e*., AltRP-expressing *M*. *smegmatis*, undergoes a complex morphogenesis in response to Zn^2+^ depletion including cell elongation, defined lipid body appearance, changes in the cell wall structure, drastic increase in size of intracellular polyphosphate bodies, and nucleoid condensation. The clear change in distribution of polyphosphate in cells from ZRM and ZLM may be the basis for the increase in phosphate content reported decades ago for *M*. *smegmatis* starved for Zn^2+^ [40]. The Δ*altRP* mutant formed well defined lipid bodies and large PPBs, similar to other strains in ZLM. However, the Δ*altRP* mutant differed from other cells grown in ZLM in that it did not form filaments with four or more polyphosphate bodies and condensed DNA. Additionally, both WT in ZRM and the Δ*altRP* mutant in ZLM had polar PPBs surrounded by a crescent of lipid rich material that was not present in the same form in the WT and Δ*altRP*/c. The differences in cellular material surrounding PPBs provide a possible explanation as to why PPBs in different strains interact differently with resin during fixation, leading to the fact that PPBs were differentially retained during cross-sectioning.

Interestingly, some of the morphological changes observed here in the context of Zn^2+^ deficiency have also been observed in *M*. *avium* treated with certain antibiotics: altered cell wall structure including enlargement of the “periplasmic space”, some in concert with condensation of the nucleoid [41]. *Burkholderia pseudomallei*, an unrelated Gram negative bacterium, forms elongated cells (filaments) with enlarged periplasmic space, which resemble the cells observed here, when exposed to ceftazidime, an antibiotic targeting the cell wall [42]. It is tempting to conclude, from the similarity with the deficiencies caused by antibiotics that the Zn^2+^-depleted morphotype observed here is also due to decreased enzymatic activities of Zn^2+^-dependent enzymes involved in cell wall formation, or other processes requiring Zn^2+^. However, if that was the case, the Δ*altRP* mutant, due to its higher demand for Zn^2+^ to build PrimRP-containing ribosomes, would enter such change sooner than WT, which is the opposite from what we observe. Therefore, these changes appear to be part of a more general stress response to Zn^2+^-depletion, which in natural environments is likely to coincide with deficiencies in other essential nutrients.

Formation of elongated *M*. *smegmatis* filaments has been previously described, but during mild nutrient starvation for carbon and nitrogen [36] and Fe^2+^ [40]. Unlike our observations, *M*. *smegmatis* filaments formed in response to carbon and nitrogen starvation were multi-septated and multi-nucleated and could further divide into multiple small resting cells. Interestingly, both these filamentous and short resting cell forms demonstrated characteristics of persistent cells: reduced metabolism, general stress resistance and increased antibiotic tolerance [36]. *M*. *tuberculosis* persister cells have been previously shown to over-express AltRPs, while down-regulating many other essential genes, including all other ribosomal proteins [43]. Certain morphological changes in the AltRP-expressing strains are associated with dormancy and/or persistence, *e*.*g*., condensed DNA and increased energy reserves (polyphosphate bodies) due to decreased metabolism. Indeed, polyphosphate metabolism in mycobacteria is implicated in persistence and virulence [44–47]. In addition, the stringent response is tied to polyphosphates in mycobacteria and is widely implicated to be involved in persister cell development and long-term survival of *M*. *tuberculosis* in the host [47,48]. Therefore, Zn^2+^-depletion triggered morphogenesis may in fact be a dormancy-related adaptation.

We observed striking similarities between the AltRP-dependent changes in mycobacterial physiology during Zn^2+^ depletion and stringent response-induced dormancy. For example, colony morphology of the Δ*altRP* mutant in *M*. *smegmatis* grown without Zn^2+^ is highly reminiscent, in both features and color (lack of pigment), to the colony morphology of a stringent response-null *relA* mutant in *M*. *smegmatis* [33]. Additionally, RelA mutants demonstrated less DNA compaction than the WT in response to nutrient starvation, similar to what we observed between the Δ*altRP* mutant and AltRP-expressing *M*. *smegmatis* strains during Zn^2+^ depletion [49]. RelA is a ribosome-dependent (p)ppGpp synthase which is activated by adopting an open conformation on stalled ribosomes, thus initiating the stringent response [50]. The first effect of RelA-driven (p)ppGpp production is accumulation of intracellular polyphosphate [44]. Moreover, DNA replication is inhibited by (p)ppGpp accumulation [51], and (p)ppGpp signaling nucleotides control cell morphology and cell division in *M*. *smegmatis* [35]. If incorporation of AltRPs into ribosomes initiates the stringent response during Zn^2+^ depletion, this could be the basis for the AltRP-dependent morphogenesis observed here.

The fact that the Δ*altRP* mutant divided at a similar rate as the WT during gradual Zn^2+^ depletion, but did not exhibit the same morphology was surprising. Perhaps PrimRP-containing ribosomes in the Δ*altRP* mutant under limited, but still adequate Zn^2+^ levels, allow normal replication and cell division, but cannot provide sufficient translational capacity required for cell elongation and other features observed in the WT. However, it is important to note that the Δ*altRP* mutant is not simply “arrested” in the morphotype observed before AltRP expression (*i*.*e*., in mid-log phase), but continues to divide and follow the trend of forming shorter cells, even though other Zn^2+^ induced morphological changes like PPB enlargement and appearance of lipid bodies do occur. These findings suggest that AltRPs are somehow involved in the process of Zn^2+^-depletion induced morphogenesis in *M*. *smegmatis*, which raises the question, could AltRPs serve a role beyond functional replacement of their Zn^2+^-dependent paralogs? The relationship between ribosome composition and bacterial physiology is not unprecedented, as studies in *Pseudomonas syringae* and *P*. *aeruginosa* showed that glutamylation of the ribosomal protein S6 regulates ribosome composition and function, having an effect on their proteome and pathogenicity [52]. Similarly, AltRP-containing ribosomes formed under Zn^2+^-limited conditions may have unique characteristics compared to their PrimRP-containing counterparts (*e*.*g*., localization, mRNA specificity, and stability) allowing for specific morphological changes and continuation of cell division and growth.

Although underappreciated in prokaryotes, ribosome heterogeneity and the role of ribosome specialization in eukaryotic physiology and human diseases has been a focus of numerous studies in recent years [53]. Our study implicates bacterial AltRPs in previously unknown function that parallels ribosome specialization seen in eukaryotes. Indeed, the morphological changes observed in *M*. *smegmatis* starved for Zn^2+^ in concert with the requirement for AltRPs, suggest that AltRP-containing ribosomes serve a specific role in the Zn^2+^-depletion triggered morphogenesis and also enable survival and growth under Zn^2+^-limited conditions.

## Supporting information

S1 Table Sequences of PrimRPs and AltRPs used for generating trees in Fig 1. (PDF)

S2 Table Zur regulon in *M. smegmatis*. (PDF)

S3 Table Ribosomal proteins identified using mass spectrometry of ribosomes isolated from 3-day old WT *M. smegmatis* culture grown in Sauton’s without added Zn^2+^ (ZLM). (PDF)

S1 Fig Alignment of PrimRPs and AltRPs in mycobacteria. Alignment of protein sequences for **(A)** S14, **(B)** S18, **(C)** L28, and **(D)** L33.
(PDF)

S2 Fig Position of PrimRPs in *M. smegmatis* ribosome. Image for 70S ribosome was obtained using the PyMOL Molecular Graphics System (Version 2.0 Schrödinger, LLC.) using PDB # 5O61 coordinates.
(PDF)

S3 Fig P_altRP_-*mCherry* reporter strain growth and fluorescence. (PDF)

S4 Fig Reduction of Alamar Blue by *M. smegmatis* strains in ZLM + TPEN with and without Zn^2+^ supplementation. (PDF)

S5 Fig Expression of genes encoding S18-1 (PrimRP) and S18-2 (AltRP) proteins. (PDF)

S6 Fig Polyphosphate bodies (PPBs) in the WT grown in HZM. (PDF)
