# Supplementary material for: Alternative ribosomal proteins are required for growth and morphogenesis of Mycobacterium smegmatis under zinc limiting conditions
Source: PLoS One. 2018 Apr 23;13(4):e0196300. doi: 10.1371/journal.pone.0196300 (PMC5912738; doi:10.1371/journal.pone.0196300)
Supplement: S1 Table — (PDF) [file pone.0196300.s001.pdf]

S1 Table. Sequences of PrimRPs and AltRPs used for generating trees in Fig. 1

RP	Strain	UniProt #	Sequence
	M. smegmatis mc^2 155 L28-1	A0QV03	MAAVCDICGKGPGFGKSVSHSHRRTSRRWNPNIQPVRAVTRPGGNKQRINACTSCIKAGKVSRA
	M. avium 104 L28-1	A0A0H2ZVU8	MAAVCDICGKGPGFGKSVSHSHRRTSRRWNPNVQTVHAVARPGGNKQRLNVCTSCIKAGKVVRG
	M. KMS L28-1	A1UEC1	MAAVCDVCGKGPGFGKSVSHSHRRTSRRWNPNIQTVRAVSHPGGNKKRVNVCTSCLKAGKVARG
	M. vanbaalenii PYR-1 L28-1	A1T718	MAAVCEICGKGPGFGKSVSHSHRRTSRRWDPNIQSVRAVAAPGGNKHRVNVCTSCLKAGKVSRG
	M. gilvum PYR-GCK L28-1	A4TE30	MAAVCEICGKGPGFGKSVSHSHRRTSRRWNPNIQSVRAVSSPGGNKQRVNVCTSCLKAGKVARG
L28-1	*M. marinum* M L28-1	B2HIJ5	MAAVCDICGKGPGFGKSVSHSHRRTSRRWDPNVQTVRAVTRPGGNKKRLNVCTSCIKAGKVSRG
	M. avium paratuberculosis L28-1	V7KT79	MAAVCDICGKGPGFGKSVSHSHRRTSRRWNPNVQTVHAVARPGGNKQRLNVCTSCIKAGKVVRG
	M. tuberculosis H37Rv L28-2	A5U6Y6	MAAVCDICGKGPGFGKSVSHSHRRTSRRWDPNIQTVHAVTRPGGNKKRLNVCTSCIKAGKITRG
	M. JLS L28-1	ABN97702*	MAAVCDVCGKGPGFGKSVSHSHRRTSRRWNPNIQTVRAVSHPGGNKKRVNVCTSCLKAGKVARG
	M. abscessus ATCC 9977 L28-1	B1MDN7	MAAVCDVCGKGPGFGKSVSHSHRRTNRRWNPNIQTVHAVTSPGGNKQRVNACTSCIKAGKVVRGA
	M. smegmatis mc^2 155 L28-2	A0R552	MSAHCQVTGRGPGFGNSVSHSHRRTPRRWDPNIQNKTYYLPSEGRRIRLRVSAKGIKVIDRDGIEAVVARMRARGEKV
	M. avium 104 L28-2	A0A0H2ZWK9	MSADCQVTGRRPSFGNAVSHSHRRTPRRWSPNIQLKTYYLPSEGRRIRLRVSAKGIKVIDRDGIEAVVARLRRHGQQI
	M. KMS L28-2	A1UME9	MSAHCQVTGRSPGFGNRVSHSHRRTRRRWRPNIQTKTYYVPSEGRRVTLRVSAKGIKVIDRDGIDAVLARIRRQGGKI
	M. vanbaalenii PYR-1 L28-2	A1TGF8	MSAHCQVTGRAPGFGNAVSHSHRRTRRRWNPNIQTKTYYLPSEGRRVKLRVSAKGIKVIDRDGIESVVARLRREGQKI
	M. bovis BCG Pasteur 1173P2 L28-2	A0A0H3M6C2	MSAHCQVTGRKPGFGNTVSHSHRRSRRRWSPNIQQRTYYLPSEGRRIRLRVSTKGIKVIDRDGIEAVVARLRRQGQRI
L28-2	*M. gilvum* PYR-GCK L28-2	A4T5Z3	MSAHCQVTGRKPGFGNAVSHSHRRTRRRWDPNIQRKTYYLPSEGRRITLRVSTKGIKIIDRDGIESVVARLRREEHKL
	M. marinum M L28-2	B2HKQ4	MSARCQVTGRTVSFGNTVSHSHRRSRRRWSPNIQLKTYYLPSENRRIKLRVSAKGIKVIDRDGIEATVARLRRAGEQI
	M. avium paratuberculosis L28-2	V7KGT5	MSAHCQVTGRTPGFGNAVSHSHRRTRRRWSPNIQLKAYYLPSEGRRIRLRVSAKGIKVIDRDGIEAVVARLRQQGQRI
	M. tuberculosis H37Rv L28-2	P9WHA9	MSAHCQVTGRKPGFGNTVSHSHRRSRRRWSPNIQQRTYYLPSEGRRIRLRVSTKGIKVIDRDGIEAVVARLRRQGQRI
	M. JLS L28-2	ABO00881*	MSAHCQVTGRSPGFGNRVSHSHRRTRRRWRPNIQTKTYYVPSEGRRVTLRVSAKGIKVIDRDGIDAVLARIRRQGGKI
	M. abscessus ATCC 9977 L28-2	B1MFN3	MELPLSAHCEVTGRSPGFGNAVSHSHRRTRRRWSPNIQLKTYYLPSEDRRIKLRVSTKGIKVIDRDGIEAVVARLRAQGRKF
L28-3	*M.tuberculosis* H37Rv L28-3	A0A0H3M9S9	MSARCQITGRTVGFGKAVSHSHRRTRRRWPPNIQLKAYYLPSEDRRIKVRVSAQGIKVIDRDGHRGRRRAARAGSAPAHFARQAGSSLRTAAIL
	M. bovis BCG Pasteur 1173P2 L28-3	P9WHB1	MSARCQITGRTVGFGKAVSHSHRRTRRRWPPNIQLKAYYLPSEDRRIKVRVSAQGIKVIDRDGHRGRRRAARAGSAPAHFARQAGSSLRTAAIL
	B. subtilis strain 168 L28	P37807	MARKCVITGKKTTAGNNRSHAMNASKRTWGANLQKVRILVNGKPKKVYVSARALKSGKVERV
**RP**	**Strain**	**UniProt #**	**Sequence**
	M. avium 104 L33-1	A0QL73	MASSTDVRPKITLACEVCKHRNYITKKNRRNDPDRLELKKYCPNCGKHQAHRETR
	M. smegmatis mc^2 155 L33-1	A0QS39	MASSTDVRPKITLACEVCKHRNYITKKNRRNDPDRLEIKKFCPNCGTHQPHKESR
	M. KMS L33-1	A1UBE0	MASSTDVRPKITLACEVCKHRNYITKKNRRNDPDRLELKKFCPNCGTHRAHKESR
	M. vanbaalenii PYR-1 L33-1	A1T4G4	MASSTDVRPKITLACEVCKHRNYITKKNRRNDPDRLEIKKFCPNCGKHQAHKESR
	M. bovis BCG Pasteur 1173P2 L33-1	A1KGB4	MASSTDVRPKITLACEVCKHRNYITKKNRRNDPDRLELKKFCPNCGKHQAHRETR
L33-1	*M.* JLS L33-1	A3PV27*	MASSTDVRPKITLACEVCKHRNYITKKNRRNDPDRLELKKFCPNCGVHRAHKESR
	M. gilvum PYR-GCK L33-1	A4T1L7	MASSTDVRPKITLACEVCKHRNYITKKNRRNDPDRLEIKKFCPNCGKHQAHKESR
	M. abscessus ATCC 9977 L33-1	B1MH92	MASSTDVRPKITLACETCKHRNYITKKNRRNDPDRLEIKKFCPNCGSHQPHKESR
	M. marinum M L33-1	B2HSG5	MASSTDVRPKITLACEVCKHRNYITKKNRRNDPDRLELKKFCRNCGSHQAHRETR
	M. avium paratuberculosis L33-1	Q73SG8	MASSTDVRPKITLACEVCKHRNYITKKNRRNDPDRLELKKYCPNCGKHQAHRETR
	M. tuberculosis H37Rv L33-1	P9WH95	MASSTDVRPKITLACEVCKHRNYITKKNRRNDPDRLELKKFCPNCGKHQAHRETR
	M. avium 104 L33-2	A0QM60	MPRNEIRPLVKLRSTAGTGYTYITRKNRRNDPDRLVLRKYDPVVRRHVDFREER
	M. smegmatis mc^2 155 L33-2	A0R551	MARNEIRPIVKLRSTAGTGYTYVTRKNRRNDPDRIVLRKYDPVLRRHVEFREER
	M. KMS L33-2	A1UME8	MARNEIRPIVKLRSTAGTGYTYVTRKNRRNDPDRLMLKKCDPVVRRHVDFREER
	M. vanbaalenii PYR-1 L33-2	A1TGF9	MARNEIRPLVKLRSTAGTGYTYITRKNRRNDPDRITLRKYDPVVRRHVDFREER
	M. bovis BCG Pasteur 1173P2 L33-2	A1KKA3	MARTDIRPIVKLRSTAGTGYTYTTRKNRRNDPDRLILRKYDPILRRHVDFREER
L33-2	*M.* JLS L33-2	A3Q6V2*	MARNEIRPIVKLRSTAGTGYTYVTRKNRRNDPDRLMLKKYDPVVRRHVDFREER
	M. gilvum PYR-GCK L33-2	A4T600	MTRNEIRPIVKLRSTAGTGYTYVTRKNRRNDPDRIVLRKYDPVVRRHVDFREDR
	M. abscessus ATCC 9977 L33-2	B1MFN2	MARNEIRPIVKLRSTAGTGYTYVTRKNRRNDPDRMVLRKYDPVVRKHVDFREER
	M. marinum M L33-2	B2HKQ3	MARNEIRPAVKLRSTAGTGYTYITRKNRRNDPDRLILSKYDPVIRKHVPFREER
	M. avium paratuberculosis L33-2	Q73TE9	MARNEIRPLVKLRSTAGTGYTYITRKNRRNDPDRLVLRKYDPVIRRHVEFREER
	M. tuberculosis H37Rv L33-2	P9WH97	MARTDIRPIVKLRSTAGTGYTYTTRKNRRNDPDRLILRKYDPILRRHVDFREER
	B. subtilis strain 168 L33-1A	Q06798	MRVNITLACTECGERNYISKKNKRNNPDRVEFKKYCPRDKKSTLHRETK
	B. subtilis strain 168 L33-1B	P56849	MRKKITLACKTCGNRNYTTMKSSASAAERLEVKKYCSTCNSHTAHLETK
	B. subtilis strain 168 L33-2	Ref. (1)**	MRVNVTLACTETGDRNYITTKNKRTNPDRLELKKYSPRLKKYTLHRETK

S1 Table. Sequences of PrimRPs and AltRPs used for generating trees in Fig. 1 (*cont.*)

RP	Strain	UniProt #	Sequence
S14-1	*M. smegmatis* mc^2 155 S14-1	A0QSG2	MAKKALVHKANKKPKFAVRAYTRCNKCGRPHSVYRKFGLCRICLREMAHAGELPGVQKSSW
	M. vanbaalenii PYR-1 S14-1	A1T4R9	MAKKALVNKANKKPKFKVRGYTRCNRCGRPHAVFRKFGLCRICLREMAHAGELPGVQKSSW
	M. bovis BCG Pasteur 1173P2 S14-1	A1KGJ8	MAKKALVNKAAGKPRFAVRAYTRCSKCGRPRAVYRKFGLCRICLREMAHAGELPGVQKSSW
	M. gilvum PYR-GCK S14-1	A4TEC4	MAKKALVNKANKKPKFKVRGYTRCNRCGRPHAVFRKFGLCRICLREMAHAGELPGVQKSSW
	M. abscessus ATCC 9977 S14-1	B1MGD4	MAKKALVNKAAKKPKFAVRAYTRCNRCGRPHAVFRKFGLCRICLREMAHAGELPGIHKSSW
	M. marinum M S14-1	B2HCT4	MAKKALVNKAARKPKFAVRGYTRCSKCGRPRAVFRKFGLCRICLREMAHAGELPGVQKSSW
	M. avium paratuberculosis S14-1	Q73S96	MAKKALVNKAARKPKFAVRGYTRCNKCGRPRAVFRKFGLCRICLREMAHAGELPGVQKSSW
	M. tuberculosis H37Rv S14-1	P9WH57	MAKKALVNKAAGKPRFAVRAYTRCSKCGRPRAVYRKFGLCRICLREMAHAGELPGVQKSSW
S14-2	*M. smegmatis* mc^2 155 S14-2	A0R550	MAKKSKIVKNEQRRELVQRYAERRAELKRTIRDPASSPERRAAAVSALQRLPRDSSPVRLRNRDVVDGRPRGHLRKFGLSRVRVREMAHRGELPGVRKASW
	M. vanbaalenii PYR-1 S14-2	A1TGG0	MAKKSKIVQNERRREIVARHAERRAELKAIIKSPSTTPEARVAAQSELNRQPRDASPVRVRNRDSVDGRPRGHLRKFGLSRVRVRELAHQGQLPGVRKSSW
	M. bovis BCG Pasteur 1173P2 S14-2	A1KKA2	MAKKSKIVKNQRRAATVARYASRRTALKDIIRSPSSAPEQRSTAQRALARQPRDASPVRLRNRDAIDGRPRGHLRKFGLSRVRVRQLAHDGHLPGVRKASW
	M. gilvum PYR-GCK S14-2	A4T5Z9	MAKNSKIVKNERRLVLVARHAERRAELKAIISSPSTPADARAAAQSELNRQPRDASPVRVRNRDAVDGRPRGHLRKFGLSRMRVRELAHRGQLPGVRKSSW
	M. abscessus ATCC 9977 S14-2	B1MFN1	MAKKSKIAKNEQRRAIVERYAERRAELKEIIRSPASSPEQRVAAQSELNRQPRDASAVRLRNRDAVDGRPRGYLRKFGLSRVRVRELVHDGSLPGVRKASW
	M. marinum M S14-2	B2HKQ2	MAKKSKIVKNNKRRDIVARYAQRRAELKQIIQSPTSSYEQRLDAQRALSRQPRDASAVRLRNRDAIDGRPRGHLRKFGLSRVRVRELAHAGQLPGVRKASW
	M. avium paratuberculosis S14-2	Q73TF0	MAKKSKIVKNDRRRATVARYAERRAELKEIIRSPRSTPEQRTAAQNELAHQPRDASAVRVRNRDAVDGRPRGHLRKFGLSRVRVRELAHAGQLPGVRKASW
	M. tuberculosis H37Rv S14-2	P9WH59	MAKKSKIVKNQRRAATVARYASRRTALKDIIRSPSSAPEQRSTAQRALARQPRDASPVRLRNRDAIDGRPRGHLRKFGLSRVRVRQLAHDGHLPGVRKASW
	B. subtilis strain 168 S14-1	P12878	MAKKSMIAKQQRTPKFKVQEYTRCERCGRPHSVIRKFKLCRICFRELAYKGQIPGVKKASW
	B. subtilis strain 168 S14-2	O31587	MAKKSKVAKELKRQQLVEQYAGIRRELKEKGDYEALSKLPRDSAPGRLHNRCMVTGRPRAYMRKFKMSRIAFRELAHKGQIPGVKKASW
**RP**	**Strain**	**UniProt #**	**Sequence**
S18-1	*M. smegmatis* mc^2 155 S18-1	A0R7F7	MAKSNKRRPAPEKPVKTRKCVFCSKKGQTIDYKDTALLRTYISERGKIRARRVTGNCVQHQRDIAVAVKNAREVALLPFGSSTR
	M. vanbaalenii PYR-1 S18-1	A1THY5	MAKSSTKRRPAPEKPVKTRKCVFCSKKGKNQDIDYKDTQLLRTYISERGKIRARRVTGNCVQHQRDIAIAVKNAREVALLPFSSSTR
	M. bovis BCG Pasteur 1173P2 S18-1	A1KEM3	MAKSSKRRPAPEKPVKTRKCVFCAKKDQAIDYKDTALLRTYISERGKIRARRVTGNCVQHQRDIALAVKNAREVALLPFTSSVR
	M. gilvum PYR-GCK S18-1	A4T4N9	MAKSSSTKRRPAPEKPVKTRKCVFCSKKGKNQVIDYKDTQLLRTYISERGKIRARRVTGNCVQHQRDIAIAVKNAREVALLPFSSSTR
	M. marinum M S18-1	B2HIB9	MAKSNKRRPAPEKPVKTRKCVFCAKKDQAIDYKDTALLRTYISERGKIRARRVTGNCVQHQRDIALAVKNAREVALLPFTSSAR
	M. avium paratuberculosis S18-1	Q744X6	MAKSNKRRPAPEKPVKARKCVFCAKKDQAIDYKDTALLRTYISERGKIRARRVTGNCVQHQRDIAIAVKNAREVALLPFTSSAR
	M. tuberculosis H37Rv S18-1	P9WH49	MAKSSKRRPAPEKPVKTRKCVFCAKKDQAIDYKDTALLRTYISERGKIRARRVTGNCVQHQRDIALAVKNAREVALLPFTSSVR
	M. abscessus ATCC 9977 S18-1	B1MMI0	MAKTTKRRPAPEKPVKTRKCAFCTKKALNIDYKDTNLLRTYISERGKIRARRVTGNCVQHQRDIAIAVKNAREVALLPFSSATR
S18-2	*M. smegmatis* mc^2 155 S18-2	A0R549	MMAVKKSRKRTAATELKKPRRNQLEALGVTTIDYKDVAVLRTFLSERGKIRSRHVTGLTPQQQRQVATAIKNAREMALLPMAGPR
	M. vanbaalenii PYR-1 S18-2	A1TGG1	MTARRREAAPAKKRRNLLKSLGIERVDYKDTSTLRQFISERGKIRSRSVTGLTVQQQRQVTTAVKTAREMALLPYPGQNPTGR
	M. bovis BCG Pasteur 1173P2 S18-2	A1KKA1	MAAKSARKGPTKAKKNLLDSLGVESVDYKDTATLRVFISDRGKIRSRGVTGLTVQQQRQVAQAIKNAREMALLPYPGQDRQRRAALCP
	M. gilvum PYR-GCK S18-2	A4T5Z8	MKRARRAAPLPKKRRNMFAQLGIERVDYKDTSTLRQFLSERGKIRSRTVTGLTVQQQRQVTIAVKNAREMALLPYPGQG
	M. marinum M S18-2	B2HKQ1	MKKKPRRTRRPIAAPATPAKKNLLDSLGVSEVDYKDISRLRTFISDRGKIRSRRVTGLTVQQQRQIATAIKNAREMALLPYPASRPQ
	M. avium paratuberculosis S18-2	Q73TF1	MPKKPARTRRPAPLAGRARKKNLLDSLGLRTVDYKDTATLRVFISERGKIRSRQVTGLSVQQQRQVATAIKNAREMALLPYPGQGIKP
	M. tuberculosis H37Rv S18-2	P9WH47	MAAKSARKGPTKAKKNLLDSLGVESVDYKDTATLRVFISDRGKIRSRGVTGLTVQQQRQVAQAIKNAREMALLPYPGQDRQRRAALCP
	M. abscessus ATCC 9977 S18-2	B1MFN0	MAPKTPRNRRAKLVDTKPAKPNLFAKLGVSSVDYKDTNTLRMFLSERGKIRSRRVTGLTVQQQRQVANAIKNAREMALLPYA
	B. subtilis strain 168 S18	P21475	MAGGRRGGRAKRRKVCYFTSNGITHIDYKDVDLLKKFVSERGKILPRRVTGTNAKYQRKLTAAIKRARQMALLPYVSGE

* NCBI accesion numbers given, no UniProt accession numbers available

** Due to a frameshift mutation, *rpmGC* (encoding L33-2 protein) is a pseudogene in the *B. subtilis* 168 lineage (1). Protein sequence with the frameshift mutation corrected is shown, as previously described (1).

1. Gabriel SE, Helmann JD. Contributions of Zur-Controlled Ribosomal Proteins to Growth under Zinc Starvation Conditions *Journal of bacteriology* 2009; 191 (19):6116–6122.
