# Supplementary material for: Alternative ribosomal proteins are required for growth and morphogenesis of Mycobacterium smegmatis under zinc limiting conditions
Source: PLoS One. 2018 Apr 23;13(4):e0196300. doi: 10.1371/journal.pone.0196300 (PMC5912738; doi:10.1371/journal.pone.0196300)
Supplement: S2 Table — (PDF) [file pone.0196300.s002.pdf]

**S2 Table.** Zur regulon in *M. smegmatis* (adapted from RegPrecise website (1))

| Locus tag | Gene Name | | Protein Name/Function |
|---|---|---|---|
| MSMEG_6048 | yciC2 | | Putative metal chaperone, involved in $Zn^{2+}$ homeostasis |
| MSMEG_4486 | znr | | $Zn^{2+}$ homeostasis transcriptional regulator Znr, ArsR family |
| MSMEG_4487 | zur | | $Zn^{2+}$ homeostasis transcriptional regulator Zur, Fur family |
| MSMEG_6068 | rpmB2* | ***altRP* operon** | 50S ribosomal protein L28-2 |
| MSMEG_6067 | rpmG2 | | 50S ribosomal protein L33-2 |
| MSMEG_6066 | rpsN2 | | 30S ribosomal protein S14-2 |
| MSMEG_6065 | rpsR2 | | 30S ribosomal protein S18-2 |
| MSMEG_6069 | yciC | | Putative metal chaperone, involved in $Zn^{2+}$ homeostasis |
| MSMEG_6070 | rpmE2 | | 50S ribosomal protein L31-2 |
| MSMEG_6047 | znuA | | $Zn^{2+}$ ABC transporter, periplasmic-binding protein ZnuA |
| MSMEG_6046 | znuC | | $Zn^{2+}$ ABC transporter, ATP-binding protein ZnuC |
| MSMEG_6045 | znuB | | $Zn^{2+}$ ABC transporter, inner membrane permease protein ZnuB |
| MSMEG_6052 | znuC2 | | $Zn^{2+}$ ABC transporter, ATP-binding protein ZnuC |
| MSMEG_6053 | bluB | | Cobalamin biosynthesis protein BluB |
| MSMEG_6054 | lamB | | Conserved hypothetical protein (LamB/YcsF) |

*Note that there are some discrepancies in naming of the ribosomal proteins and their corresponding genes in the literature. For consistency, we add suffix -2 to both gene and protein names for AltRPs. Their Zur-independent homologs, which we call primary or PrimRPs, have -1 suffix.


1. Novichkov PS, Kazakov AE, Ravcheev DA, Leyn SA, Kovaleva GY, Sutormin RA, et al. RegPrecise 3.0-a resource for genome-scale exploration of transcriptional regulation in bacteria. *BMC Genomics.* 2013;14: 745.
