# Supplementary material for: Alternative ribosomal proteins are required for growth and morphogenesis of Mycobacterium smegmatis under zinc limiting conditions
Source: PLoS One. 2018 Apr 23;13(4):e0196300. doi: 10.1371/journal.pone.0196300 (PMC5912738; doi:10.1371/journal.pone.0196300)
Supplement: S3 Table — (PDF) [file pone.0196300.s003.pdf]

S3 Table. Ribosomal proteins identified using mass spectrometry* of ribosomes isolated from 3-day old WT *M. smegmatis* culture grown in Sauton's without added Zn^{2+} (ZLM)

50S Subunit RPs	Spectral Counts
L1	622
L2	1537
L3	791
L4	545
L5	454
L6	314
L7/L12	1473
L9	285
L10	294
L11	76
L13	477
L14	268
L15	585
L16	51
L17	236
L18	734
L19	371
L20	145
L21	447
L22	413
L23	106
L24	233
L25	552
L27	127
**L28-1**	**13**
**L28-2**	**353**
L29	148
L30	35
L31	71
L32	143
**L33-1**	**3**
**L33-2**	**51**
L34	3
L35	15
L36	35

30S Subunit RPs	Spectral Counts
S1	1903
S2	612
S3	607
S4	977
S5	688
S6	280
S7	1068
S8	213
S9	759
S10	219
S11	334
S12	69
S13	460
**S14-1**	**17**
**S14-2**	**61**
S15	226
S16	407
S17	504
**S18-1**	**12**
**S18-2**	**214**
S19	371
S20	177

* Ribosomes were isolated and digested as reported previously (1) and peptides were analyzed at UC Davis Genome Center – Proteomics Core by LC-MS/MS on a Q Exactive Plus Orbitrap Mass spectrometer in conjunction Proxeon Easy-nLC II HPLC (Thermo Scientific) and Proxeon nanospray source following the Core's standard protocol. Tandem mass spectra were extracted and MS/MS analyzed by Proteome Discoverer (Thermo Scientific) and X! Tandem (The GPM, thegpm.org; version X! Tandem Alanine (2017.2.1.4)), respectively. Scaffold (version Scaffold_4.8.1, Proteome Software Inc., Portland, OR) was used to validate MS/MS based peptide and protein identifications. For each identified ribosomal protein, total spectral count is shown.

1. Prisic S, Hwang H, Dow A, Barnaby O, Pan TS, Lonzanida J a., Chazin WJ, Steen H, Husson RN. Zinc Regulates a Switch between Primary and Alternative S18 Ribosomal Proteins in *Mycobacterium tuberculosis*. *Mol Microbiol* 2015; 97:263–280.
