# Supplementary material for: Alternative ribosomal proteins are required for growth and morphogenesis of Mycobacterium smegmatis under zinc limiting conditions
Source: PLoS One. 2018 Apr 23;13(4):e0196300. doi: 10.1371/journal.pone.0196300 (PMC5912738; doi:10.1371/journal.pone.0196300)
Supplement: S1 Fig — Alignment of protein sequences for (A) S14, (B) S18, (C) L28, and (D) L33. (PDF) [file pone.0196300.s004.pdf]

S1 Fig A. S14 protein sequence alignment

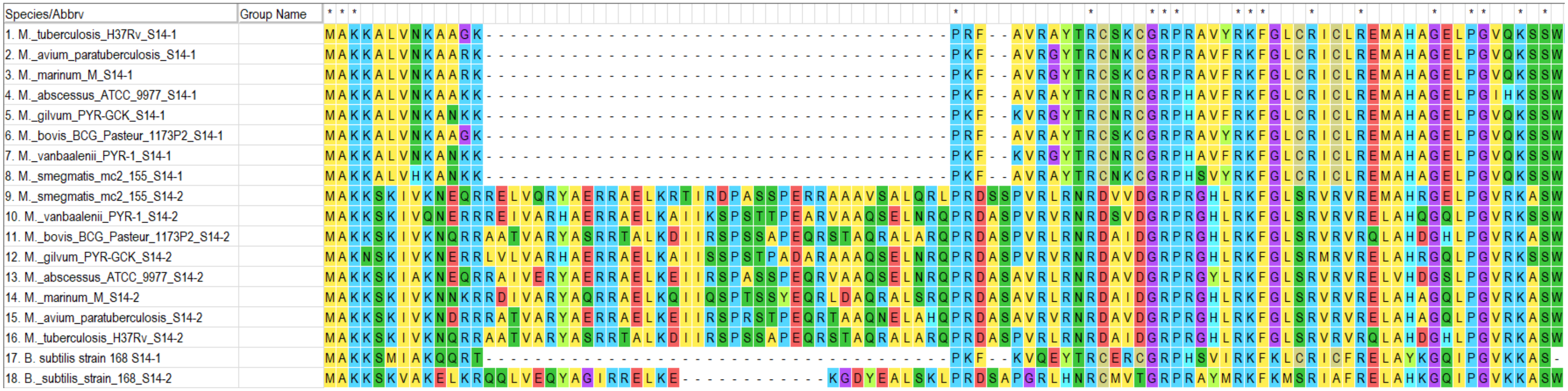

S1 Fig B. S18 protein sequence alignment

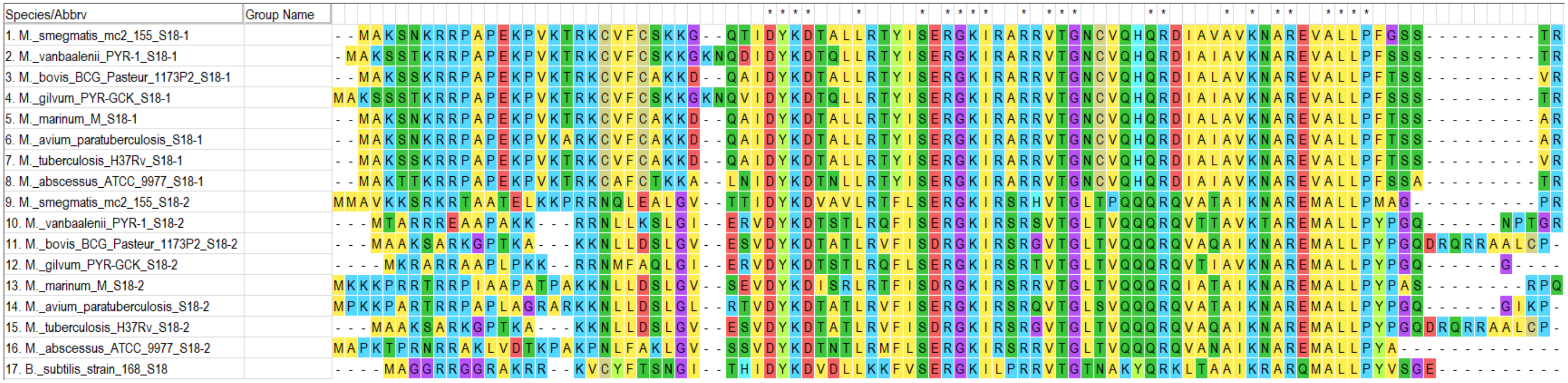

S1 Fig C. L28 protein sequence alignment

Species/Abbrv	Group Name	`        *   *     *   **      * *  * *                      *     *`
1. M._smegmatis_mc2_155_L28-1		----MAAVCDICGKGPGFGKSVSHSHRRTSRRWNPNIQPVRAVTRPGGNKQRINACTSCIKAGKVSRA--------------------------------
2. M._avium_104_L28-1		----MAAVCDICGKGPGFGKSVSHSHRRTSRRWNPNVQTVHAVARPGGNKQRLNVCTSCIKAGKVVRG--------------------------------
3. M._KMS_L28-1		----MAAVCDVCGKGPGFGKSVSHSHRRTSRRWNPNIQTVRAVSHPGGNKKRVNVCTSCLKAGKVARG--------------------------------
4. M._vanbaalenii_PYR-1_L28-1		----MAAVCEICGKGPGFGKSVSHSHRRTSRRWDPNIQSVRAVAAPGGNKHRVNVCTSCLKAGKVSRG--------------------------------
5. M._gilvum_PYR-GCK_L28-1		----MAAVCEICGKGPGFGKSVSHSHRRTSRRWNPNIQSVRAVSSPGGNKQRVNVCTSCLKAGKVARG--------------------------------
6. M._marinum_M_L28-1		----MAAVCDICGKGPGFGKSVSHSHRRTSRRWDPNVQTVRAVTRPGGNKKRLNVCTSCIKAGKVSRG--------------------------------
7. M._avium_paratuberculosis_L28-1		----MAAVCDICGKGPGFGKSVSHSHRRTSRRWNPNVQTVHAVARPGGNKQRLNVCTSCIKAGKVVRG--------------------------------
8. M._tuberculosis_H37Rv_L28-1		----MAAVCDICGKGPGFGKSVSHSHRRTSRRWDPNIQTVHAVTRPGGNKKRLNVCTSCIKAGKITRG--------------------------------
9. M._JLS_L28-1		----MAAVCDVCGKGPGFGKSVSHSHRRTSRRWNPNIQTVRAVSHPGGNKKRVNVCTSCLKAGKVARG--------------------------------
10. M._abscessus_ATCC_9977_L28-1		----MAAVCDVCGKGPGFGKSVSHSHRRTNRRWNPNIQTVHAVTSPGGNKQRVNACTSCIKAGKVVRGA-------------------------------
11. M._smegmatis_mc2_155_L28-2		----MSAHCQVTGRGPGFGNSVSHSHRRTPRRWDPNIQNKTYYLPSEGRRIRLRVSAKGIKV--IDRDG---------IEAVVARMRARGEKV-------
12. M._avium_104_L28-2		----MSADCQVTGRRPSFGNAVSHSHRRTPRRWSPNIQLKTYYLPSEGRRIRLRVSAKGIKV--IDRDG---------IEAVVARLRRHGQQI-------
13. M._KMS_L28-2		----MSAHCQVTGRSPGFGNRVSHSHRRTRRRWRPNIQTKTYYVPSEGRRVTLRVSAKGIKV--IDRDG---------IDAVLARIRRQGGKI-------
14. M._vanbaalenii_PYR-1_L28-2		----MSAHCQVTGRAPGFGNAVSHSHRRTRRRWNPNIQTKTYYLPSEGRRVKLRVSAKGIKV--IDRDG---------IESVVARLRREGQKI-------
15. M._bovis_BCG_Pasteur_1173P2_L28-2		----MSAHCQVTGRKPGFGNTVSHSHRRSRRRWSPNIQQRTYYLPSEGRRIRLRVSTKGIKV--IDRDG---------IEAVVARLRRQGQRI-------
16. M._gilvum_PYR-GCK_L28-2		----MSAHCQVTGRKPGFGNAVSHSHRRTRRRWDPNIQRKTYYLPSEGRRITLRVSTKGIKI--IDRDG---------IESVVARLRREEHKL-------
17. M._marinum_M_L28-2		----MSARCQVTGRTVSFGNTVSHSHRRSRRRWSPNIQLKTYYLPSENRRIKLRVSAKGIKV--IDRDG---------IEATVARLRRAGEQI-------
18. M._avium_paratuberculosis_L28-2		----MSAHCQVTGRTPGFGNAVSHSHRRTRRRWSPNIQLKAYYLPSEGRRIRLRVSAKGIKV--IDRDG---------IEAVVARLRQQGQRI-------
19. M._tuberculosis_H37Rv_L28-2		----MSAHCQVTGRKPGFGNTVSHSHRRSRRRWSPNIQQRTYYLPSEGRRIRLRVSTKGIKV--IDRDG---------IEAVVARLRRQGQRI-------
20. M._JLS_L28-2		----MSAHCQVTGRSPGFGNRVSHSHRRTRRRWRPNIQTKTYYVPSEGRRVTLRVSAKGIKV--IDRDG---------IDAVLARIRRQGGKI-------
21. M._abscessus_ATCC_9977_L28-2		MELPLSAHCEVTGRSPGFGNAVSHSHRRTRRRWSPNIQLKTYYLPSEDRRIKLRVSTKGIKV--IDRDG---------IEAVVARLRAQGRKF-------
22. M.tuberculosis_H37Rv_L28-3		----MSARCQITGRTVGFGKAVSHSHRRTRRRWPPNIQLKAYYLPSEDRRIKVRVSAQGIKV--IDRDGHRGRRRAARAGSAPAHFARQAGSSLRTAAIL
23. M._bovis_BCG_Pasteur_1173P2_L28-3		----MSARCQITGRTVGFGKAVSHSHRRTRRRWPPNIQLKAYYLPSEDRRIKVRVSAQGIKV--IDRDGHRGRRRAARAGSAPAHFARQAGSSLRTAAIL
24. B._subtilis_strain_168_L28		----MARKCVITGKKTTAGNNRSHAMNASKRTWGANLQKVRILV--NGKPKKVYVSARALKSGKVERV--------------------------------

S1 Fig D. L33 protein sequence alignment

Species/Abbrv	Group Name	`       *    *         *   *       *    *            *`
1. M._avium_104_L33-1		MASSTDVRPKITLACEVCKHRNYITKKNRRNDPDRLELKKYCPNCGKHQAHRETR
2. M._smegmatis_mc2_155_L33-1		MASSTDVRPKITLACEVCKHRNYITKKNRRNDPDRLEIKKFCPNCGTHQPHKESR
3. M._KMS_L33-1		MASSTDVRPKITLACEVCKHRNYITKKNRRNDPDRLELKKFCPNCGTHRAHKESR
4. M._vanbaalenii_PYR-1_L33-1		MASSTDVRPKITLACEVCKHRNYITKKNRRNDPDRLEIKKFCPNCGKHQAHKESR
5. M._bovis_BCG_Pasteur_1173P2_L33-1		MASSTDVRPKITLACEVCKHRNYITKKNRRNDPDRLELKKFCPNCGKHQAHRETR
6. M._JLS_L33-1		MASSTDVRPKITLACEVCKHRNYITKKNRRNDPDRLELKKFCPNCGVHRAHKESR
7. M._gilvum_PYR-GCK_L33-1		MASSTDVRPKITLACEVCKHRNYITKKNRRNDPDRLEIKKFCPNCGKHQAHKESR
8. M._abscessus_ATCC_9977_L33-1		MASSTDVRPKITLACETCKHRNYITKKNRRNDPDRLEIKKFCPNCGSHQPHKESR
9. M._marinum_M_L33-1		MASSTDVRPKITLACEVCKHRNYITKKNRRNDPDRLELKKFCRNCGSHQAHRETR
10. M._avium_paratuberculosis_L33-1		MASSTDVRPKITLACEVCKHRNYITKKNRRNDPDRLELKKYCPNCGKHQAHRETR
11. M._tuberculosis_H37Rv_L33-1		MASSTDVRPKITLACEVCKHRNYITKKNRRNDPDRLELKKFCPNCGKHQAHRETR
12. M._avium_104_L33-2		-MPRNEIRPLVKLRSTAGTGYTYITRKNRRNDPDRLVLRKYDPVVRRHVDFREER
13. M._smegmatis_mc2_155_L33-2		-MARNEIRPIVKLRSTAGTGYTYVTRKNRRNDPDRIVLRKYDPVLRRHVEFREER
14. M._KMS_L33-2		-MARNEIRPIVKLRSTAGTGYTYVTRKNRRNDPDRLMLKKCDPVVRRHVDFREER
15. M._vanbaalenii_PYR-1_L33-2		-MARNEIRPLVKLRSTAGTGYTYITRKNRRNDPDRITLRKYDPVVRRHVDFREER
16. M._bovis_BCG_Pasteur_1173P2_L33-2		-MARTDIRPIVKLRSTAGTGYTYTTRKNRRNDPDRLILRKYDPILRRHVDFREER
17. M._JLS_L33-2		-MARNEIRPIVKLRSTAGTGYTYVTRKNRRNDPDRLMLKKYDPVVRRHVDFREER
18. M._gilvum_PYR-GCK_L33-2		-MTRNEIRPIVKLRSTAGTGYTYVTRKNRRNDPDRIVLRKYDPVVRRHVDFREDR
19. M._abscessus_ATCC_9977_L33-2		-MARNEIRPIVKLRSTAGTGYTYVTRKNRRNDPDRMVLRKYDPVVRKHVDFREER
20. M._marinum_M_L33-2		-MARNEIRPAVKLRSTAGTGYTYITRKNRRNDPDRLILSKYDPVIRKHVPFREER
21. M._avium_paratuberculosis_L33-2		-MARNEIRPLVKLRSTAGTGYTYITRKNRRNDPDRLVLRKYDPVIRRHVEFREER
22. M._tuberculosis_H37Rv_L33-2		-MARTDIRPIVKLRSTAGTGYTYTTRKNRRNDPDRLILRKYDPILRRHVDFREER
23. B._subtilis_strain_168_L33-1A		------MRVNITLACTECGERNYISKKNKRNNPDRVEFKKYCPRDKKSTLHRETK
24. B._subtilis_strain_168_L33-1B		------MRKKITLACKTCGNRNYTTMKSSASAAERLEVKKYCSTCNSHTAHLETK
25. B._subtilis_strain_168_L33-2		------MRVNVTLACTETGDRNYITTKNKRTNPDRLELKKYSPRLKKYTLHRETK
