# Supplementary figures and images for: Alternative ribosomal proteins are required for growth and morphogenesis of Mycobacterium smegmatis under zinc limiting conditions
Source: PLoS One. 2018 Apr 23;13(4):e0196300. doi: 10.1371/journal.pone.0196300 (PMC5912738; doi:10.1371/journal.pone.0196300)

**S2 Fig. Position of PrimRPs in *M. smegmatis* ribosome**

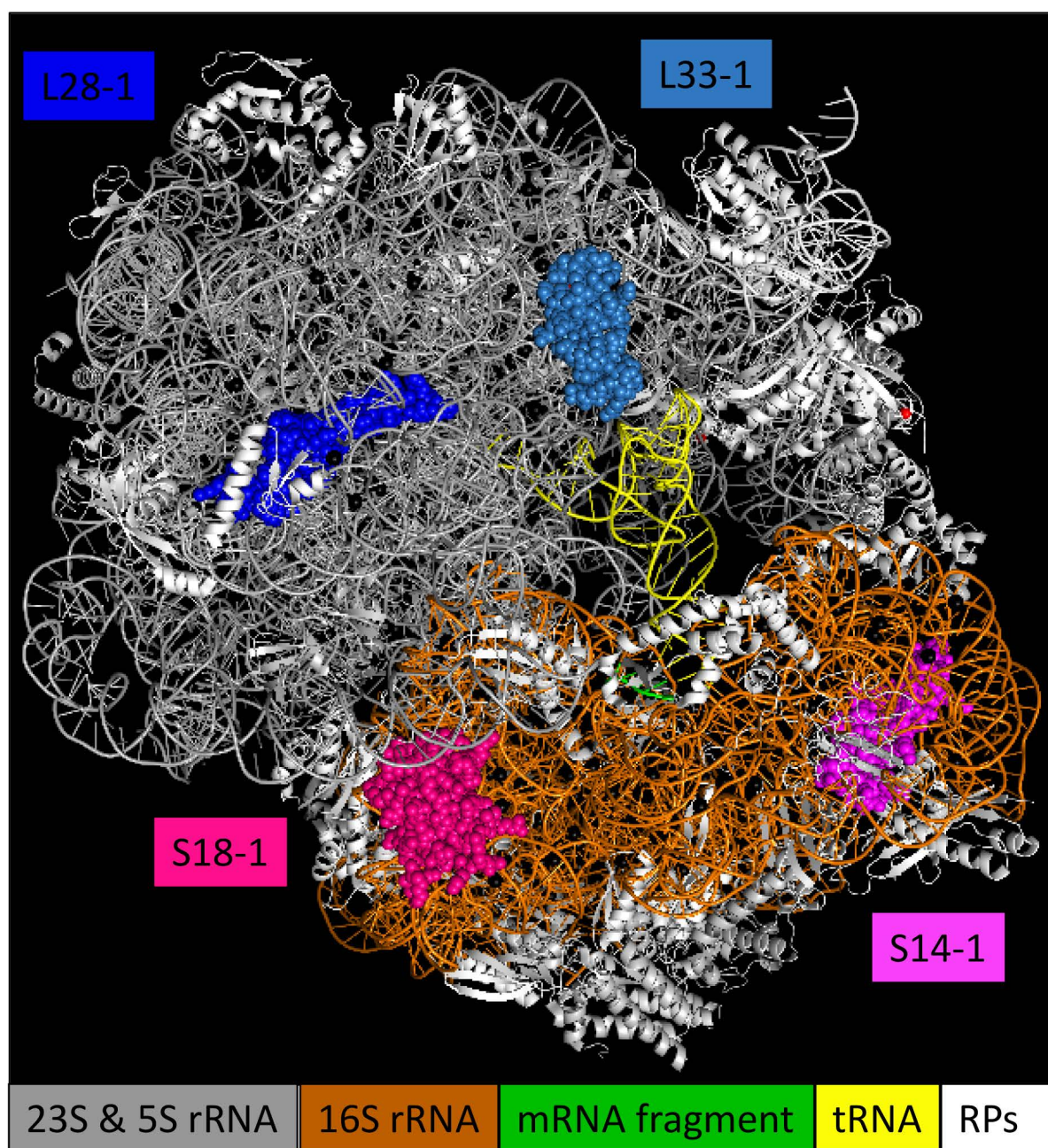

Supplement: S2 Fig — Image for 70S ribosome was obtained using the PyMOL Molecular Graphics System (Version 2.0 Schrödinger, LLC.) using PDB # 5O61 coordinates. (PDF) [file pone.0196300.s005.pdf]
