# Supplementary material for: Alternative ribosomal proteins are required for growth and morphogenesis of Mycobacterium smegmatis under zinc limiting conditions
Source: PLoS One. 2018 Apr 23;13(4):e0196300. doi: 10.1371/journal.pone.0196300 (PMC5912738; doi:10.1371/journal.pone.0196300)
Supplement: S3 Fig — (PDF) [file pone.0196300.s006.pdf]

S3 Fig. P_{altRP}-*mCherry* reporter strain growth and fluorescence

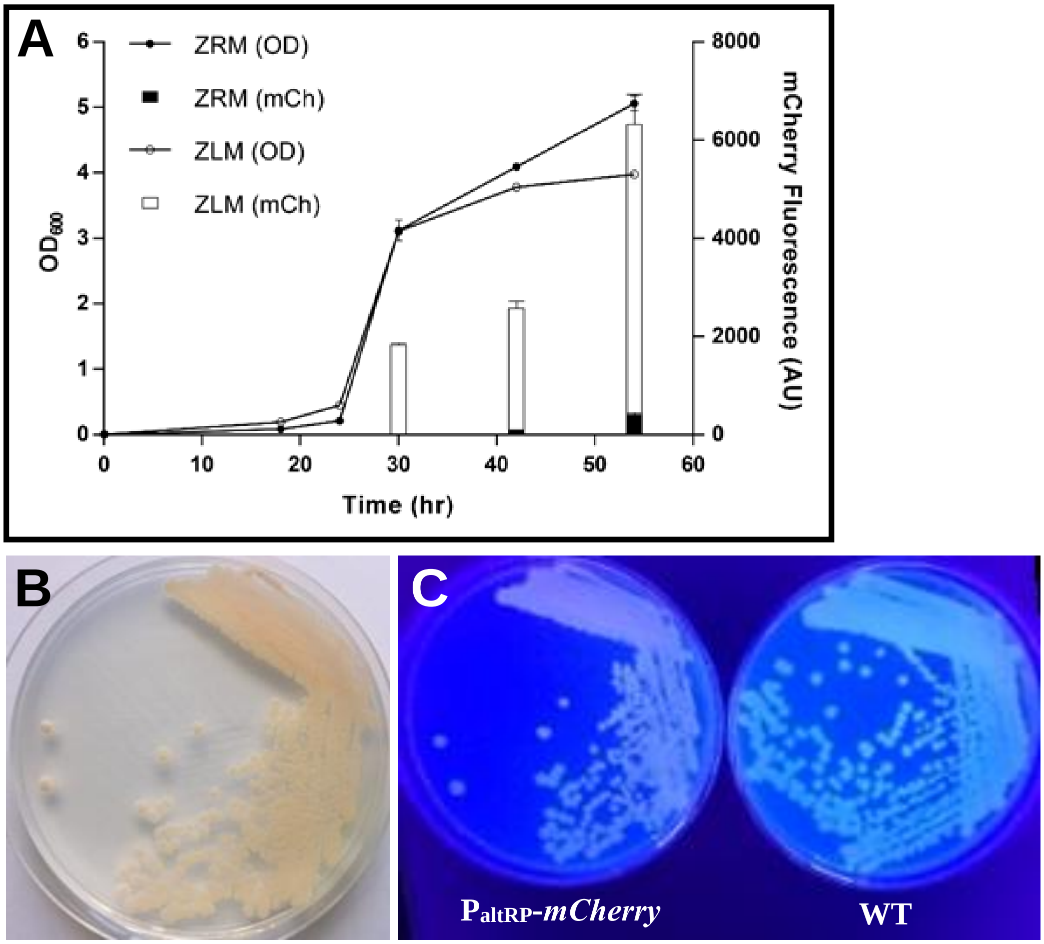

S3 Fig. Growth and fluorescence of the P_{altRP}-*mCherry* reporter strain in liquid and solid media. **(A)** Concurrent growth (left axis, lines) and fluorescence (right axis, bars) of mCherry fluorescent protein expressed from the *altRP* promoter in the P_{altRP}-*mCherry* reporter strain grown in both zinc replete (ZRM) and zinc limited (ZLM) media. This strain was grown in parallel with strains reported in Fig. 2 to signify AltRP expression. The bars representing fluorescence of cultures grown in ZRM are stacked on top of the bars representing fluorescence in ZLM. The small increase in fluorescence observed in stationary phase in ZRM is background fluorescence; the spectral properties are not consistent with mCherry fluorescence and the WT strain grown in ZRM shows the same increase in fluorescence even in absence of the reporter plasmid. Strains grown in ZLM do not demonstrate an increase in background fluorescence. Strains were grown in biological duplicate and error bars represent standard deviation. **(B)** Demonstration of pink colonies from the P_{altRP}-*mCherry* reporter strain grown on solid ZLM. Plates were incubated at 37°C for one week in the dark (to avoid carotenoid production that might interfere with observing mCherry-induced color, see text). **(C)** mCherry fluorescence from colonies of P_{altRP}-*mCherry* reporter strain is also apparent under UV light, WT did not exhibit any pink color and is shown for comparison.
