# Supplementary material for: Alternative ribosomal proteins are required for growth and morphogenesis of Mycobacterium smegmatis under zinc limiting conditions
Source: PLoS One. 2018 Apr 23;13(4):e0196300. doi: 10.1371/journal.pone.0196300 (PMC5912738; doi:10.1371/journal.pone.0196300)
Supplement: S4 Fig — (PDF) [file pone.0196300.s007.pdf]

S4 Fig. Reduction of Alamar Blue by *M. smegmatis* strains in ZLM + TPEN with and without Zn^{2+} supplementation

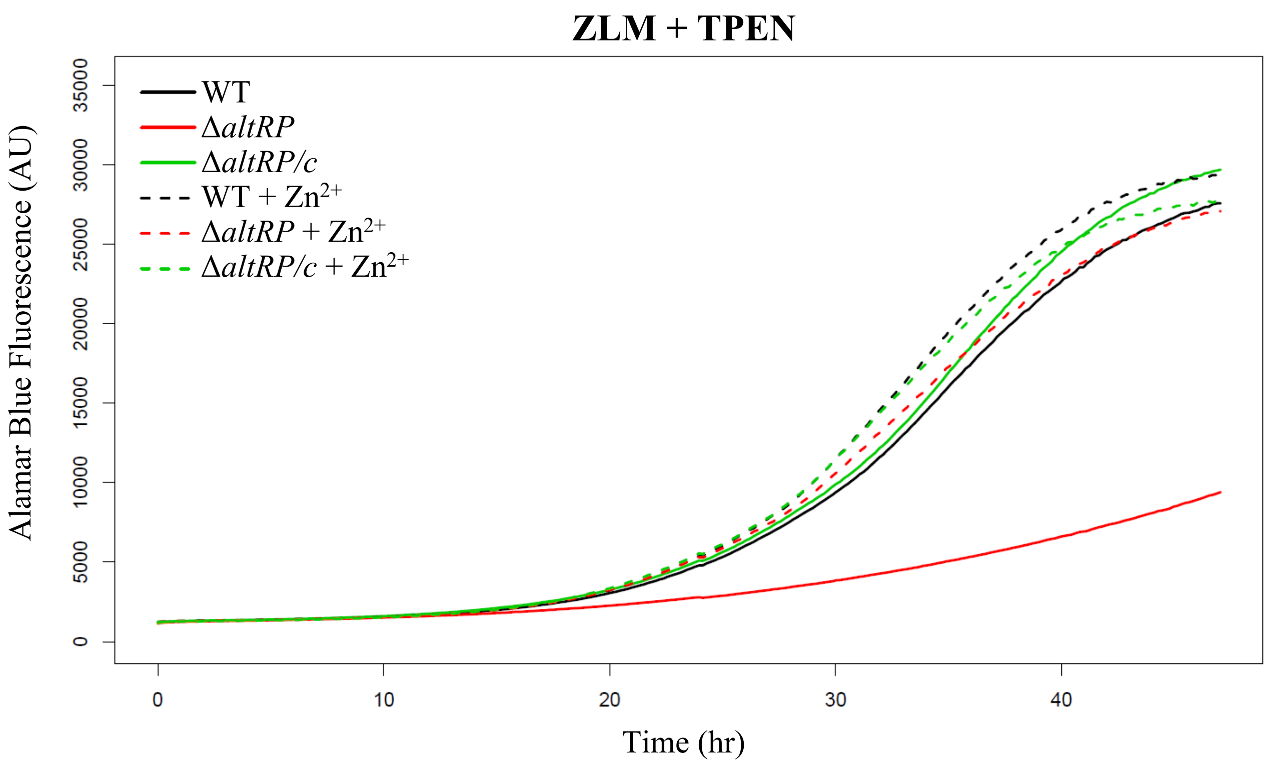

S4 Fig. Reduction of Alamar Blue® (ThermoFisher Scientific) reagent over time by WT, Δ*altRP*, and Δ*altRP/c*. Overnight cultures were grown in 7H9/ADC media as described in materials and methods section. Overnight cultures were pelleted, washed two times in ZLM and normalized to an OD_{600} of 1.0. OD1 cells were further diluted 1:500 in ZLM and 100 µL was added to wells of a 96-well plate. Additionally, 100 µL of ZLM with 1.5 µM TPEN or ZLM with 1.5 µM TPEN and 1.5 µM Zn^{2+} was added to each well, for final concentrations of 0.75 µM TPEN and Zn^{2+}. Increase in Alamar Blue® fluorescence indicates increased cellular respiration as a result of increasing cell numbers. Accumulation of reduced Alamar Blue® fluorescence (550nm/595nm) was measured every 10 minutes for two days in a Tecan plate reader. Graph represents the averages of two biological replicates. All sample means had less than 10% variance, and at the end of the experiment all wells were pink except the Δ*altRP* mutant grown in LZM + TPEN (solid red line), which were blue.
