# Supplementary material for: Alternative ribosomal proteins are required for growth and morphogenesis of Mycobacterium smegmatis under zinc limiting conditions
Source: PLoS One. 2018 Apr 23;13(4):e0196300. doi: 10.1371/journal.pone.0196300 (PMC5912738; doi:10.1371/journal.pone.0196300)
Supplement: S5 Fig — (PDF) [file pone.0196300.s008.pdf]

S5 Fig. Expression of genes encoding S18-1 (PrimRP) and S18-2 (AltRP) proteins

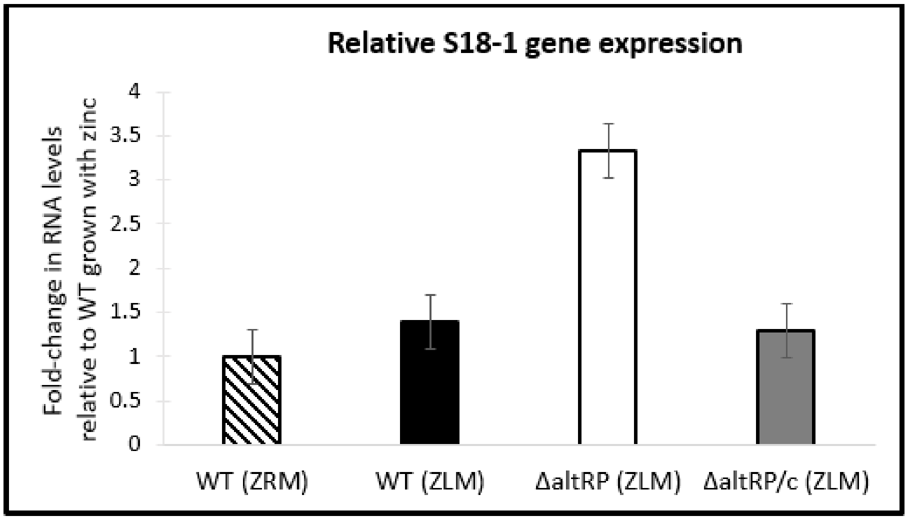

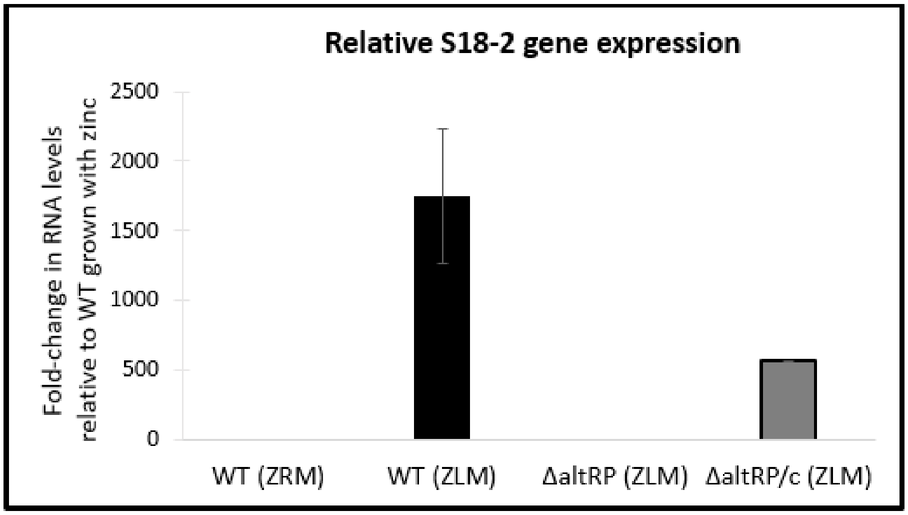

S5 Fig. Change in mRNA levels as quantified by qRT-PCR. Bacterial cultures were prepared as described in the materials and methods and harvested after 72 hours of growth in either ZRM or ZLM as indicated. RNA was extracted with TRIzol^{TM} (ThermoFisher Scientific) reagent, purified with an RNA purification column, and treated with DNAse I before being normalized to 40 ng/µL. 100 ng of RNA was used in 20 µL volume qRT-PCRs using Power SYBR® Green RNA-to-CT TM 1-Step Kit (Applied Biosystems). Genomic DNA contamination was determined by "no RT" 16S rRNA reactions to be less than 0.003% in each sample. Ct values from primers specific to the genes encoding the primary S18-1, or the alternative S18-2, ribosomal proteins were normalized to the Ct values from 16S rRNA for each sample. Results of gene expression are normalized to the expression levels of the wild type grown with Zn^{2+} (ZRM). Standard deviations represent averages of biological triplicate.
