# Supplementary material for: Alternative ribosomal proteins are required for growth and morphogenesis of Mycobacterium smegmatis under zinc limiting conditions
Source: PLoS One. 2018 Apr 23;13(4):e0196300. doi: 10.1371/journal.pone.0196300 (PMC5912738; doi:10.1371/journal.pone.0196300)
Supplement: S6 Fig — (PDF) [file pone.0196300.s009.pdf]

S6 Fig. Polyphosphate bodies (PPBs) in the WT grown in HZM

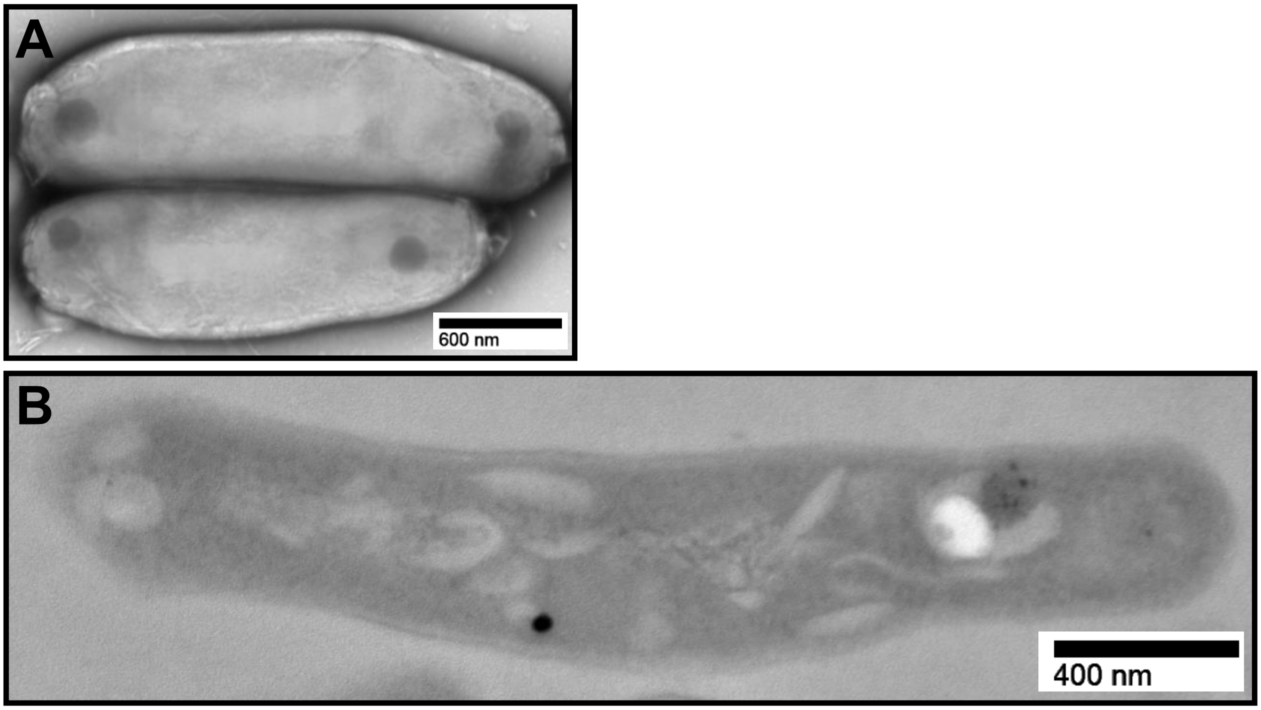

S6 Fig. Polyphosphate bodies (PPBs) in the WT grown in HZM. **(A)** Example of PPB distribution in cells grown to late stationary phase (Day 7) in HZM. **(B)** Example of a retained PPB in WT grown in HZM. Notice the PPB is still present (dark gray circle with black spots above the empty white space on the right side of the cell), but is dislodged from its original location as we observed in cells grown in LZM.
